# Zhuriheng pills improve adipose tissue dysfunction and inflammation by modulating PPARγ to stabilize atherosclerotic plaques

**DOI:** 10.3389/fphar.2025.1576521

**Published:** 2025-10-20

**Authors:** Qiong Zhai, Fangyuan Liang, Guanlin Yang, Zhimin Liu, Xin Dong, Ren Bu, Peifeng Xue, Shengsang Na, Xuan Zhang, Pengwei Zhao, Xiaoning Wang, Qiang Wei, Yuewu Wang, Jingkun Lu

**Affiliations:** 1 School of Basic Medicine, Inner Mongolia Medical University, Huhehot, China; 2 School of Pharmaceutical and Environmental Engineering, Nantong Vocational University, Nantong, China; 3 Pharmaceutical Department, Zaozhuang Thoracic hospital (Zaozhuang Cancer Hospital), Zaozhuang, China; 4 Pharmaceutical Department, Chifeng College Affiliated Hospital, Chifeng, China; 5 School of Pharmacy, Inner Mongolia Medical University, Huhehot, China; 6 Medical Innovation Center for Nationalities, Inner Mongolia Medical University, Huhehot, China

**Keywords:** Zhuriheng Pills, Atherosclerotic plaques, the adipose tissue dysfunction and inflammation, PPARγ signaling pathway, the adipose tissue dysfunction, the adipose tissue inflammation

## Abstract

**Background:**

Adipose tissue dysfunction and chronic inflammation contribute to atherosclerosis plaque development. Zhuriheng pills (ZRH) are an effective Mongolian herbal formula used in treating coronary heart disease in China, but their mechanisms of action have not been fully elucidated.

**Purpose:**

To assess whether ZRH alleviates atherosclerosis (AS) and stabilizes plaque, this study investigates the modulatory effects of ZRH on AS and adipose tissue profiles in atherosclerotic model mice with vulnerable plaque to reveal the potential mechanisms and representative quality markers (Q-markers) of ZRH.

**Methods:**

*In vivo,* the vulnerable atherosclerotic plaque model was induced in ApoE^−/−^ mice treated with intense co-stimulation. The anti-AS effect of ZRH was assessed by serum lipid profile, hematoxylin–eosin (HE), Oil O Red, Masson staining, immunohistochemistry (IHC), immunofluorescence, and plasma lipidomics. *In vitro*, a co-culture model was established with 3T3-L1 adipocytes treated with palmitic acid (PA) and RAW264.7 macrophages treated with lipopolysaccharide (LPS). The potential mechanism and Q-markers of ZRH were identified by lipid content test, inflammatory factors and adipocytokine analysis, flow cytometry for macrophage polarization, and Western blotting for PPAR*γ* and UCP-1 proteins.

**Results:**

*In vivo*, ZRH stabilizes plaques by improving serum lipid profiles, lowering macrophage infiltration, and boosting collagen content in plaques. ZRH can counteract HFD-induced adipocyte hypertrophy, increase UCP-1 and PPARγ expression, enhance the “browning” of adipose tissue, and inhibit macrophage M1 polarization. Lipidomics results showed that ZRH treatment increased the abundance of lipid species with multiple unsaturated bonds and decreased harmful TAG, DAG, and HexCer. In addition, ZRH regulates inflammatory factors and adipokines in co-culture to inhibit macrophage M1 polarization and adipocyte abnormal lipid metabolism. In contrast, RDK and its monomers have a stronger anti-inflammatory effect, whereas GZ and its monomers regulate lipid metabolism better. ZRH was shown to be a PPARγ agonist for improving adipose tissue dysfunction and inflammation for anti-AS effects of ZRH. MIX, which comprises ellagic acid (G3), quercetin (G8), 3,3′-Di-O-methylellagic acid (G9), elemicin (R5), and safrole (R8) in equal proportions, is only one of ZRH’s Q-markers. More research is needed on the roles of different ZRH metabolites.

**Conclusion:**

Our results demonstrated that ZRH stabilizes atherosclerotic plaques by ameliorating adipose tissue dysfunction and inflammation by regulating the PPARγ pathway.

## Introduction

Atherosclerosis (AS) is a hallmark of atherosclerotic cardiovascular disease (ASCVD) and the major contributor to cardiovascular disease (CVD) mortality. AS is closely related to issues such as abnormal lipid dysregulation, chronic inflammation, and endothelial dysfunction*.* Acute cardiovascular events are mostly caused by thrombus from the erosion of vulnerable plaques (Libby and Hansson, 2019; Naghavi et al., 2003). Macrophages, the dominant immune cells within AS plaques, participate in multiple processes including the formation, progression, rupture, and healing of atherosclerotic plaques, *via* their plasticity of polarizing states and function (Hou P, et al., 2023). Furthermore, according to multiple studies, there is a positive correlation between coronary heart disease (CHD) risk and abnormal adipose tissue. In particular, inflammation, metabolic disorder, and the lowered “browning” thermogenic capacity of adipose tissue are directly linked to endothelial dysfunction and inflammatory cell infiltration in the blood vessels. As adipose tissue as a complex endocrine organ, excessive storage of triglycerides in fat tissue can trigger local and systemic inflammatory responses and interfere with the secretion of adipokines and macrophage polarization, thus further accelerating atherosclerosis development (Rigano et al., 2017; Singh et al., 2021; Mu W et al., 2021).

Adipose tissue plays an important role in regulating whole-body energy metabolism through its storage function in white adipocytes (WAT) and its dissipating function in brown (BAT) and beige adipocytes (Kita et al., 2019). Dysfunctional adipose tissue promotes a pro-inflammatory, hyperlipidemic, and insulin resistant environment (Chait and den Hartigh, 2020). In adipose tissue, adipocytes and macrophages participate in the pro-inflammatory feedback cycle in adipose tissue, leading to chronic inflammation (Balakumar et al., 2019).

Peroxisome proliferator-activated receptors (PPARs) are not only essential regulators of whole-body metabolism but also modulate inflammation in immune cells, notably macrophages (Lefere et al., 2020). PPARs include PPARα, PPARβ/δ, and PPARγ. Of these, PPARγ is mostly located in adipose tissue and the immune system; it is a crucial mediator between adipose tissue and atherosclerosis, controlling glucose and lipid metabolism, lipogenesis, and inflammatory response (Welch et al., 2022; Qian J and Sun Y, 2021). PPAR activators, such as PPARγ agonists, have been used for metabolic disorders and improving cardiovascular outcomes (Wang and Zhang, 2022).

In China, the prescription of Zhuriheng drip pill (ZRH) originated from a Mongolian clinical formula for the treatment of cardiovascular diseases. The herbal medicine composition of ZRH includes Fructus Choerospondiatis (seed) (emperor, GuangZao, GZ), *Myristica fragrans* Houtt*.(seed)* (minister, RouDoukou, RDK), Lignum AquilariaeResinatum (ChenXiang, CX), Amomum Tsao-ko (CaoGuo, CG), asafetida (AWei, AW), *Carthamus tinctorius L.* (HongHua, HH), *Gardenia jasminoides* Ellis (ZhiZi, ZZ), synthetic borneol (BingPian, BP), and Bovis Calculus Artifactus (Artificial NiuHuang, NH) (Wang et al., 2018). In our previous studies, we used rapid high-performance liquid chromatography-tandem quadrupole-electrostatic field orbitrap high-resolution mass spectrometry (HPLC-Q-Exactive-MS/MS) to identify 60 metabolites in ZRH, which were speculated in positive and negative ion modes (Zhang et al., 2024); 15 inherent metabolites were quantitatively analyzed (Bu et al., 2023). The network pharmacology, molecular docking, and spectrum-effect study of ZRH found that PPARs are the co-target of multiple metabolites which inhibit macrophage foaming by regulating the PPAR pathway (Zhai et al., 2024; Mu et al., 2019). However, despite ZRH efficacy in treating CHD, the molecular mechanisms and candidate Q-markers of ZRH towards CHD remain unclear.

Therefore, this study aims to investigate the effects of ZRH on plaque stability and adipose tissue inflammation in AS. We established an ApoE^−/−^ mouse model with atherosclerotic instability plaques (Ma et al., 2013) *in vivo* and a macrophage–adipocyte co-culture model *in vitro* to explore the underlying mechanisms of the ZRH. Our findings revealed that the ZRH pill positively impacted adipose tissue inflammation and plaque stability, validating the association between PPARγ regulation and ZRH. Meanwhile, we used an exclusion method to verify the role of the emperor and minister in prescription, improve the understanding of the compatibility mechanisms, and help identify the Q-markers of ZRH for CHD treatment.

## Materials and methods

### Drug preparation

ZRH pills contain nine botanical drugs: Choerospondiatis Fructus (dried and ripe fruits of *Choerospondias axillaris* (Roxb.) B. L. Burtt and A. W. Hill, GZ, emperor, LOT No. 1901010519) and *M. fragrans* Houtt (dried semen of *M. fragrans* Houtt, RDK, minister, LOT No. 19092603), Carthami Flos (dried flowers of *C. tinctorius L.*, LOT No. 200302), Gardeniae Fructus (dried and ripe fructus of *G. jasminoides* J. Ellis, LOT No. 19122903), Aquilariae Lignum Resinatum (dried lignum of *Aquilaria sinensis* (Lour.) Spreng, Carthami Flos*,* LOT No. 2101011040), Fructus Tsaoko (dried and ripe fruits of *Amomum tsao-ko* Crevost and Lemarié, LOT No. 018190201), Ferulae Resina (resin of *Ferula fukanensis* K. M. Shen, LOT No. 20210524AW-AW), synthetic borneol (LOT No. 19122711), and Bovis Calculus Artifacts (a combination of cholic acid, tauronic acid, bilirubin, cholesterol and trace elements, LOT No. 200501) at a ratio of 10:6:4:3:1:4:1:0.5:0.5. All botanical drugs were purchased from the Inner Mongolia Beiyu Pharmaceutical Co. Ltd. (Hohhot, China) and were identified by Dr. Peifeng Xue from the Inner Mongolia Medical University (Hohhot, China). The specimens were deposited at the herbarium of medicinal plants (Center for New Drug Safety Evaluation and Research, Inner Mongolia Medical University, GZ20180016, RDK20180017, CX20180,018, HH20180019, CG20180020, ZZ20180021, AW20180022, NH20180023, BP20180024). ZRH (LOT No. D22001) was obtained from the Baotou Traditional Chinese Medicine Co., Ltd. in China. The preparation deleting GZ (DeGZ) was made according to ZRH formula without GZ, just as the preparation lacking RDK (DeRDK) was created by excluding RDK from the nine botanical drugs. A mixture of Choerospondiatis Fructus, Gardeniae Fructus, and Carthami Flos (18:6:8) was crushed to yield a powder and then extracted twice in 70% ethanol (1:10, mass to volume ratio) under reflux conditions for 2 h each. This extract was then filtered and concentrated to prepare a final concentration equivalent to 1.02–1.03 g/mL under reduced pressure. Next, synthetic borneol and Bovis Calculus Artifactus (ratio 1:1) were added. A mixture of Myristicae Semen, Ferulae Resina, Aquilariae Lignum Resinatum, and Fructus Tsaoko (12:2:2:8) was ground to powder and passed through a 2-mm mesh, after which a GKSFE220-50-6L supercritical carbon dioxide instrument (Jiangsu, China) was used to extract the oil from the powder. Liquefied CO_2_ was pumped into the extraction vessel to maintain an extraction pressure of 28.5 MPa with a flow rate of 17 L/h. The extraction temperature was set to 41 °C, and extraction was performed for 2 h. The full extract was then mixed with polyethylene glycol 4,000 (1:2, mass ratio) and 4% polysorbate 80 to yield the final ZRH preparation.

Ellagic acid (purity, 98.1%, LOT No. 111959-201903), gallic acid (purity, 98.1%, LOT No. 110831-201906), succinic acid (purity, 98.1%, LOT No. 110896-201602), dehydrodiisoeugenol (purity, 98.1%, LOT No. 111838-201403), eugenol (purity, 98.1%, LOT No. 110725-201917), methyleugenol (purity, 98.1%, LOT No. 111642-200301), myristicin (purity, 98.1%, (LOT No. 190180-201701), and macelignan (purity, 98.1%, LOT No. 111844-201603) were purchased from the National Institute for the Control of Pharmaceutical and Biological Products (Beijing, China). Elemicin (purity, 98.1%, LOT No. 48711-6) and 3,3′-Di-O-methylellagic acid (purity, 98.1%, LOT No. DE002001) were purchased from Aoke Biology Research Co. Ltd. (Beijing, China). Safrole (purity, 98.1%, LOT No. SM190418-06), quercetin (purity, 98.1%, LOT No. 20230725), licarin A (purity, 98.1%, LOT No. 51020-87-2), and citric acid (purity, 98.1%, LOT No. DW003102) were purchased from WeiyeJL Technology Research Institute (Beijing, China). Catechin (purity, 98.1%, LOT No. DETDE000101) was purchased from Lemeitian Pharmaceutical Technology Co., Ltd. (Chengdu, China).

### Preparation of ZRH rat intestinally absorbed solution (IAS)

Adult male Sprague–Dawley rats from SPF Biotechnology Co., Ltd. in Beijing, China, aged 6–8 weeks, were used in the everted intestinal sac tests. A drug-containing intestinally absorbed solution (IAS) was obtained as described by Zhai et al. (2024) with a rat everted gut sac (EGS) model. After 0.252 g/mL De-GZ, 0.302 g/mL De-RDK, 0.378 g/mL ZRH, or 20 mL of K-R buffer were separately perfused in thr gut sacs for 2 h, the “Blank-IAS,” “De-GZ-IAS,” “De-RDK-IAS,” and “ZRH-IAS” solutions in the sacs were collected and stored at −80 °C for *in vitro* studies.

### Quality control analysis of ZRH-IAS

Methanol was used to prepare individual stock solutions (1 mg/mL) of 14 analytes and IS of digoxin. The stock solutions were serially diluted with Blank-IAS to obtain a mixed standard working solution. Six batches of ZRH-IAS or Blank-IAS samples were accurately mixed with the standard solution and an IS of digoxin solution with ratio 10:5:1, respectively. Protein was extracted and precipitated with methanol, vortexed, and centrifugated, and a 200-μL aliquot of the supernatant was dried. Before determination, the residue was reconstituted with methanol and filtered. For quality control for ZRH-IAS, we employed rapid high performance liquid chromatography tandem quadrupole-electrostatic field orbitrap high resolution mass spectrometry (HPLC-Q-Exactive-MS/MS, Thermo Fisher Scientific Inc., Grand Island, United States). Chromatography was performed on an ACE Excel 3 C18-PFP column (3 × 100 mm, 3 µm). A gradient of methanol containing 0.2% formic acid (solvent A) and water containing 0.2% formic acid (solvent B) was used for the elution procedure: 0.5 min, 5% A; 1 min, 10% A; 1min, 15% A; 1min, 20% A; 1 min, 25% A; 1.5 min, 30% A; 3.5 min, 40% A; 2 min, 45% A; 3 min, 52% A; 1.5 min, 62% A; 1.5 min, 67% A; 1.5 min, 70% A; 1.5 min, 80% A; 0.5 min, 80%–2% A; 3 min, 2% A. The column temperature and flow rate were set at 35 °C and 0.3 mL·min^−1^, respectively. From each sample, 5 µL was injected into the column.

HPLC-Q-Exactive-MS/MS analysis coupled with an electrospray ionization (ESI) source was performed in full scan/dd ms2 mode. The MS parameters were provided as follows: sheath gas flow rate, 35 L/min; aux gas flow rate, 10 L/min; spray voltage, 2.8 kV for negative mode and 3.5Kv for positive mode; capillary temperature, 350 °C; S-lens RF level, 50 kV; aux gas heater temperature, 150 °C; mass range recorded at 100–1,000 m/z.

### Animals and treatments

The study followed all institutional and national guidelines, and the experimental protocols were approved by the Ethics Committee of the Inner Mongolia Medical University in China (YKD2019146). Male ApoE^−/−^ mice (8 weeks old, 22–25 g) were purchased from SiPeiFuBioTech Co. Ltd. (Beijing, China). They were maintained in an environment with a 12-h light cycle, controlled temperature (21°C–24 °C), and free access to water and food. After 1 week of acclimation, ApoE^−/−^ mice received a high-fat diet (HFD) (BEIJING KEAO XIELI Co., Ltd., #D12079B, China) containing 21% fat, 50% carbohydrate, and 20% protein for 18 weeks, while control ApoE^−/−^ mice were fed a standard chow diet (3% calories from fat). Following 8 weeks of HFD feeding, the ApoE^−/−^ mice fed the HFD were randomly separated into five groups for administration. The dosage for ZRH is 2.8 g/day for a 70 kg patient. The treatment group was administered 368 mg/kg of ZRH (low-dose, ZRH-L), 736 mg/kg (middle-dose, ZRH-M), and 1,472 mg/kg (high-dose, ZRH-H) for 3 weeks continuously and then halted for 1 week. Simvastatin was given to the positive control group at 2.6 mg/kg, while 0.5% carboxymethylcellulose sodium (CMC-Na) was given to the control and model groups until the end of the test (*n* = 10 per group).

**FIGURE 1:**
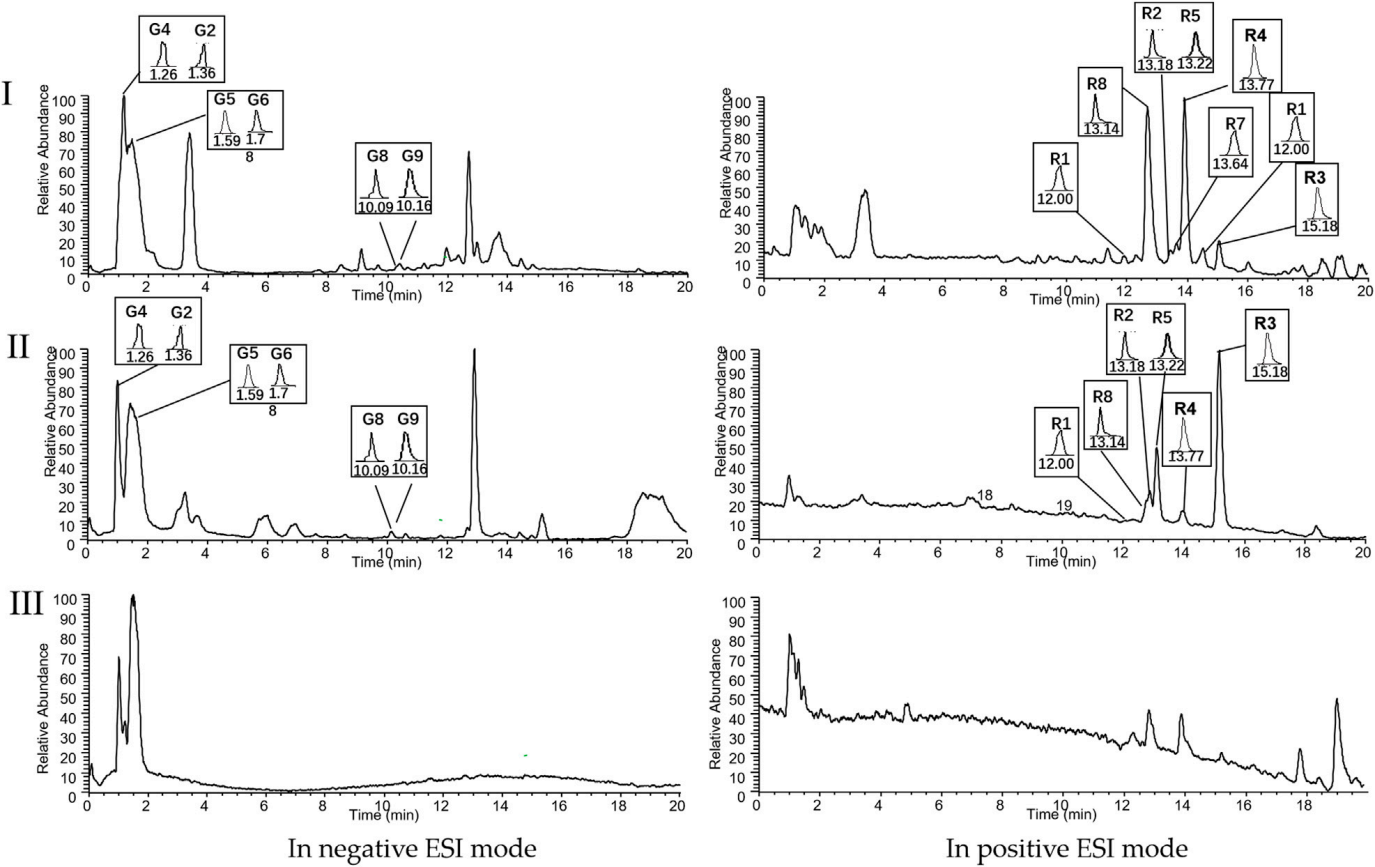
Chromatograms of (I) ZRH15-IAS, (II) QC and (III) Blank-IAS analyzed by HPLC-Q-Exactive-MS/MS. Peaks: citric acid (G2), gallic acid (G4), quercetin (G8), and 3,3′-Di-O-methylellagic acid (G9) in the negative ion mode, and eugenol (R1), methyleugenol (R2), dehydrodiisoeugenol (R3), myristicin (R4), elemicin (R5), and safrole (R8) in the positive ion mode. Blank-IAS: blank intestinal absorbed solution. ZRH15-IAS: drug-containing intestinal absorbed sample collected after 37.8 mg·mL^−1^ ZRH treatment for 2 h, QC: blank intestinal absorbed sample spiked with standards.

After 18 weeks of the HFD diet, the ApoE^−/−^ mice in the drug intervention groups were stimulated with ice-cold water (0 °C, non-submerged) for 5–10 min and injected intraperitoneally with 1 μg/kg/day of LPS over 2 days, followed by 8 μg/kg phenylephrine injection on day 3 (Figure 1A) to trigger unstable atherosclerotic plaques (Ma et al., 2013). Throughout the study period, body weight and food intake were recorded every other week for all mice.

After 24 h post-combined stimulation, all animals were euthanized. Blood samples were collected from the orbital plexus for lipidomics analysis and biochemical detection before euthanasia. After left ventricle perfusion with 4% polyformaldehyde and saline, the heart’s aortic root and the whole aorta were removed and immobilized in a 4% paraformaldehyde solution for more than 48 h. Brown adipose tissue (BAT) was found on the scapular surface, epididymal white adipose tissue (eWAT) around the epididymis in the viscera, inguinal white adipose tissue (iWAT) in the subcutaneous inguinal position, and PVAT around the abdominal aorta. They were immobilized in a paraformaldehyde solution or frozen at −80 °C for further analysis.

### Measurement of serum lipid concentrations

The concentrations of serum lipids, comprising total cholesterol (TC), triglycerides (TG), low-density lipoprotein (LDL), and high-density lipoprotein (HDL), were measured using commercially available assay kits (Nanjing Jiancheng Bioengineering Institute, Nanjing, China).

### Determination and evaluation of atherosclerotic plaques

In the case of atheroma lesion evaluations, the aortas of the mice were stained with Oil Red O (#1003091610, Sigma, GRE) to quantify the area occupied by atherosclerotic lesions, and the degree of severity was quantified with the percentage of the atherosclerotic lesion area corrected by the total aortic area. Following dehydration in a gradient of 10%, 20%, and 30% sucrose-saturated solutions, the frozen hearts’ aortic roots were serially sectioned at 7-μm using a freezing microtome (CM3050S, Leica, GRE), hematoxylin and eosin (H&E) staining was used to analyze areas of plaque and necrotic cores, and the cross-sectional area of the aortic arch was calculated. Oil Red O staining, Masson’s three-color stain (#G1340, Solarbio, China), and immunohistochemistry were used to analyze the plaque composition, which separately indicated the contents of lipids, collagens, vascular smooth muscle cells (SMC), and macrophages. An anti-α-SMA Rabbit pAb (α-smooth muscle actin; #GB111364 1:200; Servicebio, China) and an anti-F4/80 Rabbit pAb (#GB113373, 1:200; Servicebio, China) were used in this study. The plaque stability score was developed by comparing the ratio of the plaque metabolites to the whole plaque; the vulnerability index of plaque = (macrophage area + lipid area)/(SMC region + collagen region) (Lu et al., 2019; Ma et al., 2013).

### Histological analysis of adipose tissue

Embedded in paraffin wax, the fatty tissues were sliced to 5 microns and stained with H&E. The slices were fixed in 4% paraformaldehyde for 15 min, washed with PBS for 20 min, and infiltrated with 0.1% Triton X-100 for 10 min. To reduce nonspecific binding, slides were cultured in 10% diluted rabbit serum at room temperature for 60 min. These segments were then incubated with UCP1 antibodies (GB112174, 1:200; Servicebio, CHN).

### Immunofluorescence analysis of PVAT

Following dehydration in a gradient, the frozen mouse thoracic aortic PVAT was sectioned at 7-μm. For immunofluorescence (IF) staining, sections were washed with PBS three times, heated with citrate buffer for antigen retrieval, permeabilized with 0.1% Triton X100 for 10 min, and blocked with 5% goat serum at room temperature for 2 h. Primary antibodies against CD86 (ab119857; 1:500, Abcam, United Kingdom) and CD206 (GB113497, 1:500, Servicebio, CHN) were incubated overnight at 4 °C. After rinsing thrice in PBS, the sections were treated with dual fluorescently labeled secondary antibodies: Alexa-Fluor CY3-conjugated goat anti-rat (GB21302, 1:500, Servicebio, CHN) and Alexa-Fluor 488-conjugated goat anti-rabbit (GB113497, 1:500, Servicebio, CHN), then counterstained with DAPI. The sections were photographed with a microscope (Laica, DM4300 BLED) and analyzed with Image-Pro Plus 6.0.

### Sample preparation for lipidomics analysis

For lipidomics analysis, the plasma samples were sent to Novogene Technology Co., Ltd. (Beijing, China). Vortex treatment was performed on a 100-μL plasma sample (IAS) mixed with 750 μL methanol. Next, 2.5 mL of methyl tertiary buty1 ether (MTBE) was added and incubated for 1 h, adding 0.625 mL MS water to induce phase separation. After incubation for 10 min, the sample was centrifuged for 10 min at 12,000 rpm. After collection of the top phase, the bottom grade was extracted again with 1 mL of the solvent mixture (MTBE/methanol/water (10:3:2.5, v/v/v)) and the top stage was harvested. The organic phases were combined and dried. Subsequently, the residue was stored at −80 °C for less than 3 months until re-dissolved in 100 μL isopropanol for UHPLC-MS/MS analysis (Narváez-Rivas and Zhang, 2016). Quality control (QC) samples were prepared by pooling and equilibrating aliquots from each plasma sample. Plasma lipidomic ultra-high-performance liquid chromatography coupled to tandem mass spectrometry (UHPLC-MS/MS) analysis was conducted using a high-field quadrupole orbitrap (Q Exactive™ HF-X) mass spectrometer system (Thermo Fisher Scientific Inc., Dreieich, Germany), coupled with a Thermo Vanquish™ UHPLC system (Thermo Fisher Scientific Inc., Dreieich, Germany) equipped with a heated electrospray ionization interface (ESI) (UHPLC-Q-Exactive-MS/MS analysis). Chromatographic separation was performed on a Thermo Accucore C30 (2.1 × 150 mm, 2.6 µm) chip (Thermo Fisher Scientific Inc., Dreieich, Germany). Data were captured and analyzed using Xcalibur 3.1 (Thermo Fisher Scientific Inc., Dreieich, Germany). The column temperature was set at 40 °C, the flow rate was 0.35 mL/min, and the injection volume was 5 µL. The mobile phase consisted of phases A (acetonitrile/H_2_O, 6/4) and B (isopropanol/acetonitrile, 1/9) containing 0.1% formic acid and 10 mM ammonium acetate in a 20 min gradient detection as follows: 30% B, initial; 30% B, 2 min; 43% B, 5 min; 55% B, 5.1 min; 70% B, 11 min; 99% B, 16 min; 30% B, 18.1 min. Analyses were performed in both positive and negative ionic modes and a mass range recorded at 114–1700 m/z. The raw data were imported into Compound Discoverer 3.01 (CD3.1, Thermo Fisher Inc., Dreieich, Germany) for preliminary screening. The parameters set were: Rt deviation of 0.2 min; m/z deviation of 5 ppm; signal strength deviation of 30%; signal to-noise ratio of 3, minimum signal intensity of 10^5^; peak extraction of information such as addition ions. Subsequently, the molecular ion peaks and fragmentation ions predicted the molecular formula. After spectra alignment, grouping, compound detection, and matching with the m/z Cloud, m/z Vault, and Lipidmaps (https://lipidmaps.org) and Lipid Blast databases (https://fiehnlab.ucdavis.edu/projects/LipidBlast) with CD software, a multi-dimensional peak table, including the predicted formula, accurate m/z, retention time, peak area, identified lipid metabolites, and relative quantitative results, were established. Blank samples were used to remove background interference. Principal components analysis (PCA) and partial least squares discriminant analysis (OPLS-DA) were performed using LINT, a lipidmic data processing website (www.lintwebomics.info). Volcano plots and heat maps were plotted. Lipid metabolites with VIP >1 and *P* < 0.05 and fold change ≥2 or FC ≤ 0.5 were identified as differentiated. Pathway analyses of significantly different metabolites were performed using the MetaboAnalyst 5.0 (https://www.metaboanalyst.ca), and the pathways having impact values > 0.05 and −log(p) > 2.0 were analyzed.

### Cell culture and treatment

Mouse RAW264.7 macrophage and 3T3-L1 preadipocyte were obtained from the National Collection of Authenticated Cell Cultures (Shanghai, China). RAW264.7 cells were cultured in Dulbecco’s modified Eagle medium (DMEM, Gibco, C11995500BT) supplemented with 10% FBS (PAN, st30-3302). A quantity of 5.5 × 10^5^ cells/mL of RAW264.7 cells per well were cultured for 24 h. To trigger the inflammatory response, 100 μg/mL of LPS was added into RAW264.7 cells for another 24 h. We cultured 3T3-L1 cells in DMEM supplemented with 10% FBS and 1% penicillin/streptomycin (Gibco, 15140122). Confluent 3T3-L1 cells were grown in DMEM supplemented with 7% FBS and DMI (0.5 mM IBMX +1 µM dexamethasone +10 μg/mL insulin) for 4 days, then changed to DMEM containing 7% FBS and 10 μg/mL insulin for 2 days, and further cultured for another 2 days with DMEM (10% FBS) to differentiate into mature adipocytes (López-Alcalá et al., 2024; Muscarà et al., 2019). On day 8, to establish a model of lipid accumulation, 0.2 mM PA was added for 24 h. For treatment, the cells were treated with Blank-IAS, ZRH-IAS, DeGZ-IAS, and DeRDK-IAS diluted 500×, 50 µM representative monomers of ZRH (including citric acid (G2), ellagic acid (G3), gallic acid (G4), succinic acid (G5), catechin (G6), quercetin (G8), 3,3′-Di-O-methylellagic acid (G9), eugenol (R1), methyleugenol (R2), dehydrodiisoeugenol (R3), myristicin (R4), elemicin (R5), Licarin B (R6), macelignan (R7), and safrole (R8)—Table 1), 50 µM MIX (comprising G3, G8, G9, R5, and R8 in equal proportions), 10 µM rosiglitazone, or 10 µM GW9662 for 24 h, in either conditioned media (CM) or co-culture, respectively.

**TABLE 1:** Results of HPLC-Q-Exactive-MS/MS analysis of ZRH-IAS.

Nodes	Tested metabolite	Molecular formula	Selective ion	m/z	*t*_*R*_ (min)	CAS number	Concentration in ZRH-IAS (mg/mL)	Botanical drug
G2	Citric acid	C_7_H_6_O_5_	[M-H]^−^	169.01	1.36	149-91-7	24.695 ± 1.330	GZ
G3^#^	Ellagic acid	C_14_H_6_O_8_	[M-H]^−^	301.00	3.73	476-66-4	!!	GZ
G4	Gallic acid	C_6_H_8_O_7_	[M-H]^−^	191.02	1.26	77-92-9	2673.130 ± 29.158	GZ
G5	Succinic acid	C_4_H_6_O_4_	[M-H]^−^	117.01	1.59	110-15-6	—	GZ
G6	Catechin	C_7_H_6_O_4_	[M-H]^−^	153.02	1.78	8,001-48-7	—	GZ
G8^#^	Quercetin	C_15_H_9_O_7_	[M-H]^−^	301.04	10.09	117-39-5	0.284 ± 0.015	GZ
G9^#^	3,3′-Di-O-methylellagic acid	C_16_H_10_O_8_	[M-H]^−^	329.03	10.16	2239-88-5	514.141 ± 17.327	GZ
R1	Eugenol	C_10_H_12_O_2_	[M + H]^+^	165.10	12.00	97-53-0	102.770 ± 7.197	RDK
R2	Methyleugenol	C_11_H_14_O_2_	[M + H]^+^	179.11	13.18	93-15-2	0.786 ± 0.054	RDK
R3	Dehydrodiisoeugenol	C_20_H_22_O_4_	[M + H]^+^	327.16	15.18	2680-81-1	22.296 ± 0.276	RDK
R4	Myristicin	C_11_H_12_O_3_	[M + H]^+^	193.09	13.77	607-91-0	40.560 ± 1.987	RDK
R5^#^	Elemicin	C_12_H_16_O_3_	[M + H]^+^	209.12	13.22	487-11-6	1.448 ± 0.084	RDK
R6	Licarin C	C_22_H_26_O_5_	[M + H]^+^	371.19	14.47	23518-30-1	—	RDK
R7	Macelignan	C_21_H_26_O_6_	[M + Na]^+^	397.16	13.64	107534-93-0	—	RDK
R8^#^	Safrole	C_10_H_10_O_2_	[M + H]^+^	163.08	13.14	94-59-7	11.711 ± 0.302	RDK

Node: ZRH-IAS, ZRH-containing intestinally absorbed solution; GZ, *choerospondiatis fructus*; RDK, *myristica fragrans houtt*; “#”,metabolites of the MIX; “—”, qualified but not quantified metabolites; “!!”, the inherent component of ZRH; but not found in ZRH-IAS.

### Co-culture and treatment

Cell co-culture was carried out indirectly and directly. In the indirect co-culture, to explore the influence of abnormal lipid metabolism on the macrophage, RAW264.7 cells were treated with 100 μg/mL LPS and adipocyte-conditioned medium (3T3-CM), which was the supernatant of the mature adipocytes treated without 0.2 mM palmitic acid (PA), with PA or PA + drug treatment for 24 h. In the direct co-culture, a transwell system (0.4 μm pore size, Costar, Kennebunk, MA United States) was used to evaluate the effect of the inflammation microenvironment on adipocytes, and 3T3-L1 cells were grown and differentiated in the lower well. At day 8, RAW264.7 cells were grown in the upper well, followed by co-culture for 24 h without 0.2 mM PA + 100 μg/mL LPS, with PA + LPS or PA + LPS + drug treatment. Adipocytes or macrophages without co-culture served as controls. The treatment was the same as described previously.

### ELISA

After the cells were treated, the culture media were collected and cytokines, such as IL-10(1211002, DAKEWE, CHN), IL-1β (1210122, DAKEWE, CHN), TNF-α (1217102, DAKEWE, CHN), and COX-2 (E-EL-M0959, Elabscience, CHN) in the RAW264.7cell, and the levels of leptin (E-ELM0002c, Elabscience, CHN), adiponectin (E-ELM3008c, Elabscience, CHN), and COX-2 in the 3T3-L1 adipocytes were analyzed using ELISA kits, according to the manufacturer’s protocol. Serum IL-1β was also detected.

### NO assay

After treatment, the media of RAW264.7 cells were transferred and mixed with Griess reagent (ratio 1:1). Following incubation, absorbance was measured at 540 nm using a microplate reader (Multiskan FC, Thermo Fisher, United States).

### Oil red O staining and triglyceride measurement in 3T3-L1 adipocytes

The 3T3-L1 adipocytes were fixed with 4% polyformaldehyde and stained with a working solution of Oil Red O for 30 min. After removing the staining solution, the lipid accumulation was observed under a microscope (DM2500, Leica, Germany). Subsequently, the stained Oil Red O was dissolved in isopropyl alcohol and quantified at 540 nm with the microplate reader (Multiskan FC, ThermoFisher, United States). After the adipocytes were harvested, triglyceride content was measured utilizing a commercial kit (E1025-105, Applygen, CHN). The results were adjusted based on cellular protein content.

### Flow cytometry analysis

To evaluate the polarization status in RAW264.7 macrophages, the cells in various groups were harvested after rinsing with PBS containing 2% FBS and centrifugating at 450 g for 5 min. The cells were further resuspended in 500 μL binding buffer and incubated with CD86 (E-AB-F0094D; 1:100, Elabscience, CHN) and CD206 (E-AB-F1135E; 1:100, Elabscience, CHN) primary antibodies in the dark at 37 °C for 30 min. The cells were then analyzed using a flow cytometer (BD FACSCalibur, US).

### Western blotting

Western blot analyses were performed as described in Zhang et al. (2023). The 3T3-L1 adipocytes or frozen adipose samples were extracted for 30 min with RIPA lysis buffer (1% PMSF, 2% protease inhibitors, 2% phosphatase inhibitors). Proteins were separated *via* sodium dodecyl sulfate-polyacrylamide gel electrophoresis and transferred onto polyvinylidene difluoride membranes, then incubated with primary antibodies of anti- PPARγ (GB11164, 1:1,000, Servicebio, CHN), UCP-1(GB112174, 1:200; Servicebio, CHN), and GAPDH (GB12002, 1:2,000; Servicebio, CHN) overnight at 4 °C, followed by incubation with secondary antibodies (GB23303, 1:2,000; Servicebio, CHN). The membranes were treated with ECL Plus Western Blotting Detection Reagent (G2014, Servicebio, CHN) and visualized by chemiluminescence (FUSION Solo; PEQLAB Biotechnologie GmbH, Erlangen, Germany).

### Statistical analysis

Image-Pro Plus 6.0 software (Media Cybernetics, United States) was used to analyze the histopathological images. Analysis was performed with GraphPad Prism 9.0 (GraphPad Software, United States) unless otherwise indicated. Data are expressed as mean ± SD. One-way ANOVA followed by Dunnett’s test was utilized to compare differences between multiple groups. An unpaired two-tailed Student’s t-test or Wilcoxon rank sum test was used to compare the two groups. *P* < 0.05 was considered statistically significant. Partial least squares discriminant analysis (PLS-DA) and hierarchical clustering analysis (HCA) were performed with R-language heat map packages.

## Results

### Quality control of ZRH-IAS

HPLC-Q-Exactive-MS/MS analysis was used to examine the chemical profiles of ZRH-IAS. In accordance with the quality control procedures in the Pharmacopoeia of the People’s Republic of China (2020) and compared with reference standard metabolites, the ten metabolites of ZRH-IAS were validated. The reference standards and extracted-ion chromatograms of ZRH-IAS are depicted in Figure 1. The method validation for quantitative analysis is given in Supplementary Figure S1 and Supplementary Tables S1–S4. The HPLC-Q-Exactive-MS/MS/MS analysis of ZRH-IAS is presented in Table 1. A total of 14 metabolites in ZRH-IAS were identified, and ten were quantified: G2, G4, G8, and G9 in GZ, R1, R2, R3, R4, R5, and R8 in RDK. Notably, G3, an inherent metabolite of ZRH 0.905 mg/g (Bu et al., 2023), was not found in ZRH-IAS, indicating that G3 may be methylated to G9 in IAS. Methylation is an important phase II metabolism of a drug in which methylases oxidize hydroxyl groups to ether groups in the weak alkaline environment of the intestinal tract to facilitate its metabolism. Therefore, for cellular experiments, both G3 and G9 were included as representative monomer study subjects.

### ZRH ameliorates the level of serum lipids in Aop E^−/−^ mice treated with HFD and co-stimulation

**FIGURE 2:**
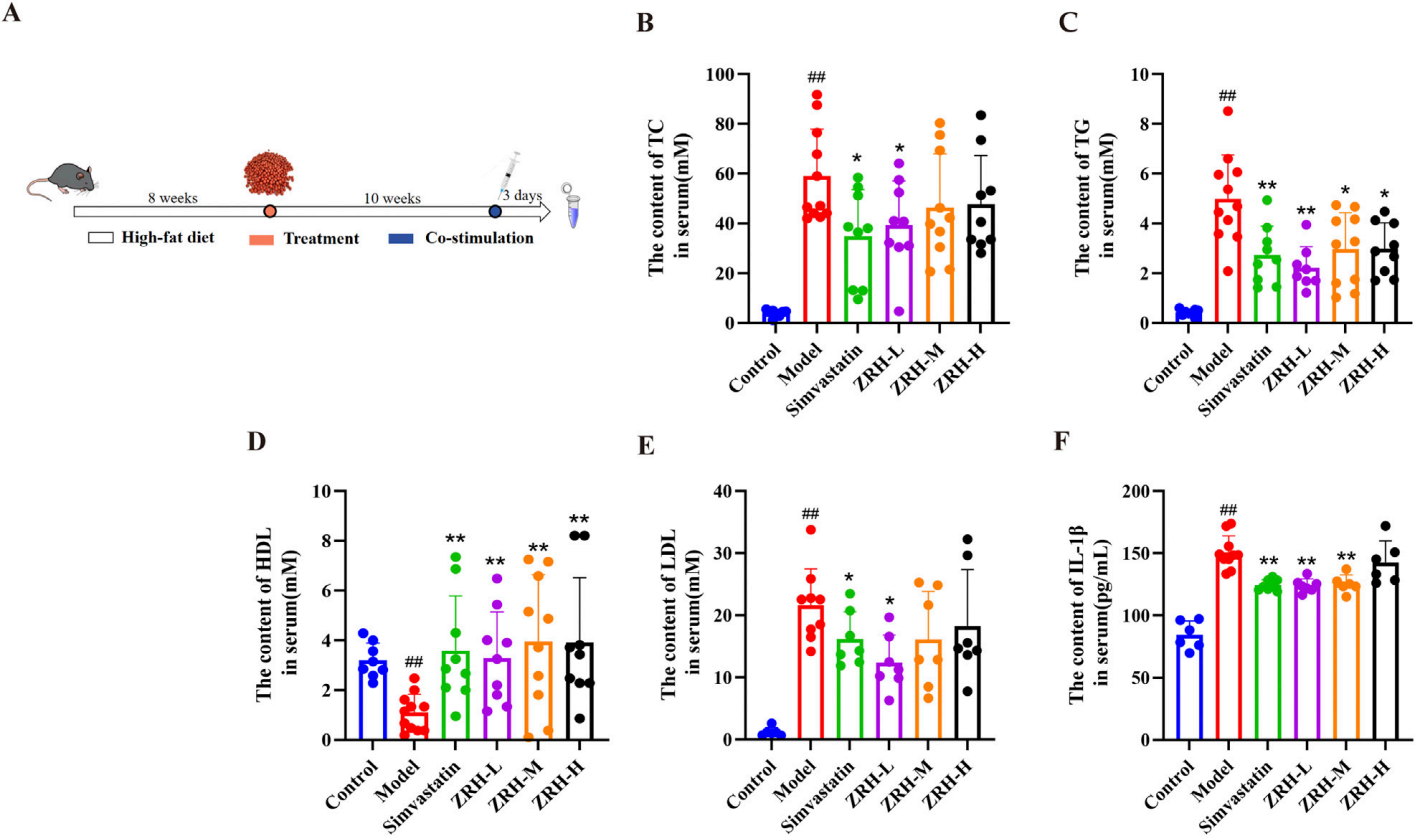
ZRH treatment ameliorated the level of serum lipids in ApoE^−/−^ mice. **(A)** Schematic representation of animal treatment. **(B–E)** Serum TC, TG, HDL, and LDL content. **(F)** Serum IL-1β concentration. TC, total cholesterol; TG, total triglyceride; HDL, high-density lipoprotein; LDL, low-density lipoprotein; IL-1β, interleukin-1β. All data presented as mean ± SD. ^#^ *P* < 0.05, ^##^ *P* < 0.01 vs. control group; *P < 0.05, **P < 0.01 vs. model group; *n* = 7-11.

Firstly, as ApoE^−/−^ mice fed an HFD along with combined stimulation are prone to building susceptible atherosclerotic plaque, we examined the impact of ZRH on body weights (Supplementary Figures S2A–F) and biochemical markers in these mice (Ma et al., 2013). The serum biochemical parameters of mice are presented in Figures 2B–E. Compared to the control group, mice in the model group had abnormal blood lipid profiles, including TC, TG, LDL, and HDL. Interestingly, after drug treatment, the mice’s hyperlipidemia was drastically reduced, with lower TG and higher HDL. Simvastatin and ZRH-L groups had the lowest TC and LDL, respectively. The model mice had greater serum IL-1β levels than control, and ZRH and simvastatin dramatically reduced IL-1β levels (Figure 2F).

### ZRH increases atherosclerotic plaque stability in ApoE^−/−^ mouse model with vulnerable plaques

**FIGURE 3:**
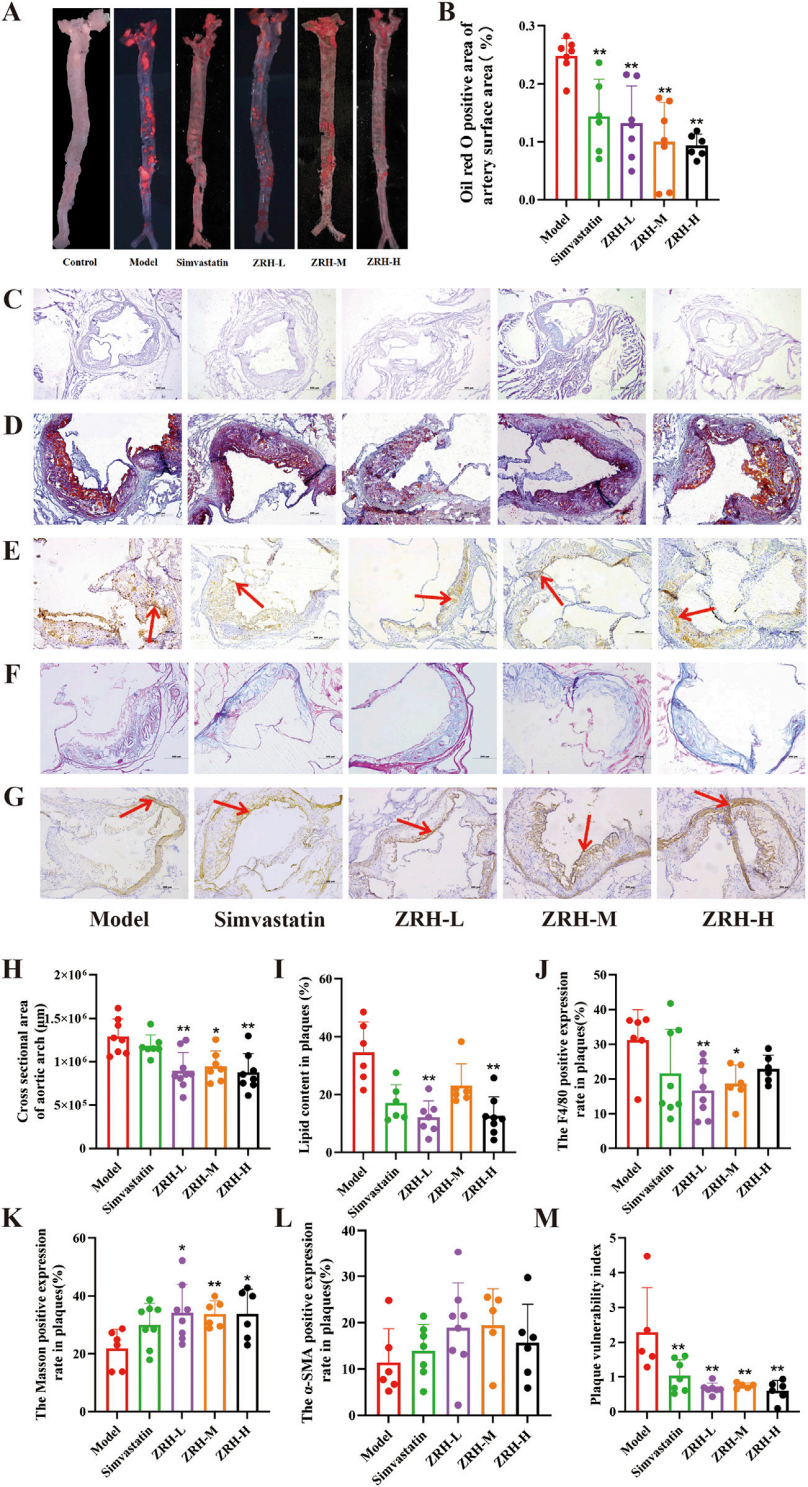
ZRH treatment reduced plaque instability in atherosclerotic mice. **(A,B)** Representative photographs and quantification of plaque burdens on the en face of the entire aorta stained with Oil-red-O. Representative images and quantification of aortic root sections stained with **(C,H)** hematoxylin and eosin (H&E), scale bar = 500 μm; **(D,I)** Oil-red-O, Scale bar = 200 μm; **(G,L)** α-smooth actin (α-SMA) immunostaining for SMCs, scale bar = 200 μm; **(E,J)** antiF4/80 immunostaining for macrophages, scale bar = 200 μm; **(F,K)** Masson’s trichrome staining for collagen, scale bar = 200 μm. **(M)** Vulnerable index of plaque = (macrophage area + lipid area)/(SMC area + collagen area). All data presented as mean ± SD.*P* < 0.05, *P < 0.01 vs. model group; n = 5–9.

Next, we investigated the anti-atherosclerotic effects of ZRH in ApoE^−/−^ mice. Oil Red O staining of the entire aortic tree revealed that the model mice showed significantly more plaque formation than control. Meanwhile, mice treated with ZRH and simvastatin showed notable decreases in lesion area (Figures 3A,B). Furthermore, cross-sectional analysis of the aortic root using H&E staining demonstrated that the development of aortic root lesions was considerably prevented in ZRH-treated animals (Figures 3C,H), strongly supporting ZRH’s atheroprotective capability *in vivo*.

To evaluate plaque stability, we analyzed the composition of the plaques, including the proportions of macrophages, lipids, smooth muscle cells (SMCs), and collagen, to calculate the vulnerability index of the plaques. In contrast to the model group, the ZRH treatment group showed increased collagen deposition, as demonstrated by Masson staining, decreased lipid content and α-SMA expression of macrophages in plaques, and a nonsignificant downward trend in α-SMA expression of SMCs (Figures 3D–G,I–L). In addition, the treatment groups’ plaque vulnerability index was lower than that of the model group, and the group differences were not very noticeable (Figure 3M). These data indicate that ZRH treatment effectively stabilizes atherosclerotic plaques by modifying key compositional characteristics.

### ZRH regulates the browning of the adipose tissue in ApoE^−/−^ mouse model with vulnerable plaques

**FIGURE 4:**
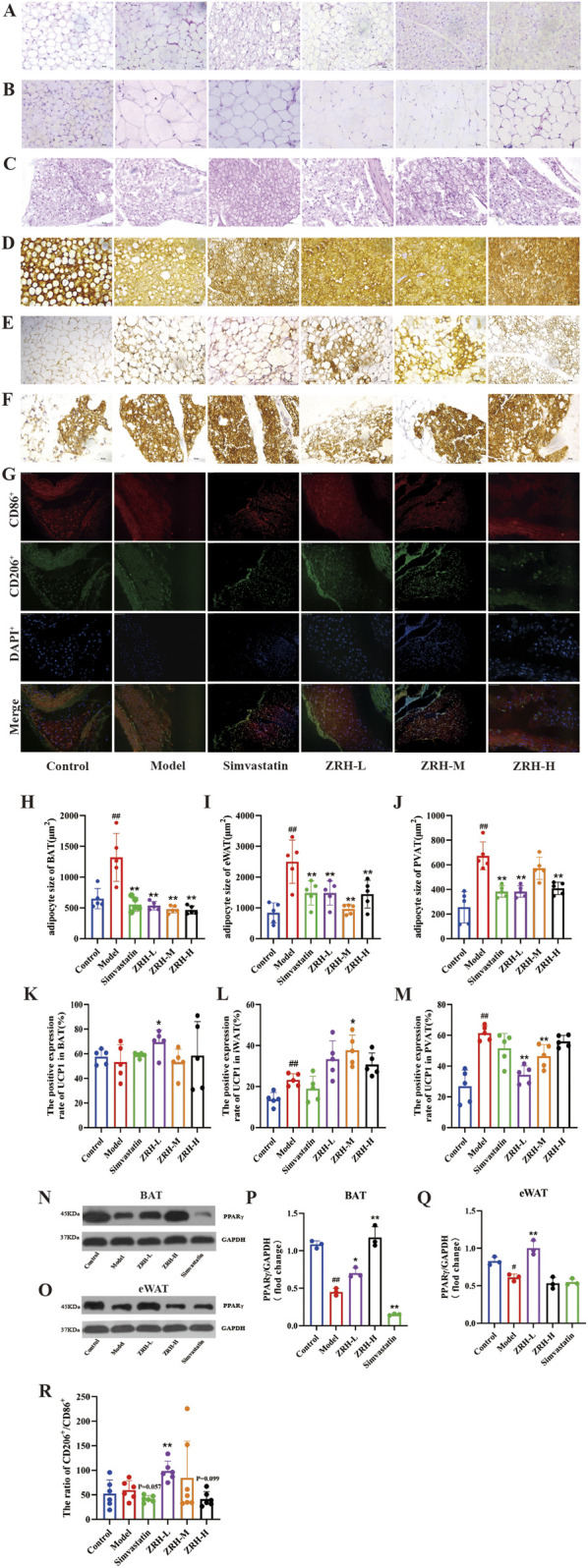
ZRH promoted the browning of the adipose tissue in atherosclerotic mice. Representative H&E staining images and quantification of **(A**,**H)** brown adipose tissue; **(B**,**I)** epididymal adipose tissue and **(C**,**J)** perivascular adipose tissue, scale bar = 50 μm. Representative immunohistochemistry images and quantification of UCP-1 expression in **(D**,**K)** brown adipose tissue, **(E**,**L)** epididymal adipose tissue, and **(F**,**M)** perivascular adipose tissue, scale bar = 50 μm. **(N–Q)** Protein expression of PPARγ in **(N**,**P)** brown adipose tissue (BAT) and **(O**,**Q)** epididymal adipose tissue (eWAT). Representative immunofluorescence staining images and quantification of perivascular adipose tissue **(G**,**R)**, scale bar = 200 μm. All data presented as mean ± SD. ^#^ *P* < 0.05, ^##^ *P* < 0.01 vs. control group; *P < 0.05, **P < 0.01 vs. model group; *n* = 5.

To explore the regulatory effect of ZRH on adipose tissue, H&E staining and UCP-1 expression with immunohistochemistry were used to observe the adipose tissue changes, and CD206 and CD86 expression was used to assess the macrophage polarization status in adipose tissue with immunofluorescence. As shown in Figures 4A–C, the H&E staining results showed that the adipocytes of the control group had a uniform size and round-like morphology. Compared to the corresponding control group, adipocytes in the eWAT, BAT, and PVAT of the model group displayed evident hypertrophy, and ZRH and simvastatin diminished the size of adipocytes (Figures 4A–C,H–J). As a result of the co-stimulation, the UCP-1 immunohistochemistry positive expression rate of the model group was higher in iWAT and BAT than in control. The data demonstrated that UCP-1 expression was markedly higher in the BAT, iWAT of ZRH-M, and BAT of the ZRH-L group than in the model group. However, UCP-1 expression was unexpectedly lower in the PVAT of the ZRH-L and ZRH-M groups than that in the model group (Figures 4D–F,K–M). Based on immunofluorescence results (Figures 4G,R), there was no appreciable variation in the ratio of CD206^+^/CD86^+^ expression between the model and control groups. The ratio dramatically increased in response to ZRH-L administration (*P* < 0.01). PPARγ regulates lipid metabolism and participates in the browning of white adipose tissue by upregulating signature thermogenic genes such as UCP-1 and PPARγ cofactor 1α (PGC-1α) (Coelho et al., 2016; Zhong et al., 2023). Further tests indicated that HFD feeding and co-stimulation markedly lowered PPARγ expression in BAT and eWAT. In contrast, PPARγ in BAT was upregulated significantly by ZRH-L and ZRH-H treatment (Figures 4N–Q) and downregulated by simvastatin. ZRH-L treatment boosted PPARγ expression in eWAT but not ZRH-H or simvastatin. These findings suggest that ZRH upregulated PPARγ expression to promote adipose tissue browning and macrophage M2 polarization in PVAT. Improving adipose tissue status may be one of ZRH’s important mechanisms against AS.

### ZRH ameliorates the plasma lipidomics profile in the ApoE^−/−^ mouse model with vulnerable plaques

**FIGURE 5:**
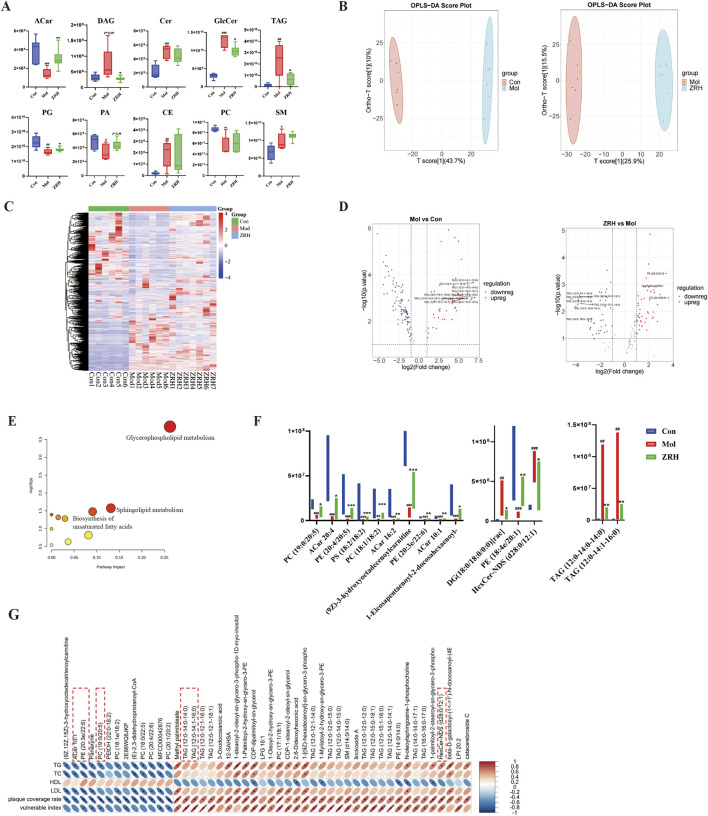
ZRH ameliorates the plasma lipidomics profile. **(A)** Chart of plasma lipid classes. **(B)** OPLS-DA of plasma between control and model groups, model and ZRH groups. **(C)** HCA heatmaps and **(D)** volcano plot showing the differences in metabolites between control and model groups, model and ZRH groups. **(E)** Enrichment analysis of significantly different metabolites (impact value > 0.05 and –log(p) > 2). **(F)** Levels of 15 potential lipid biomarkers affected by ZRH treatment. **(G)** Correlation analysis between AS-related factors and lipidomics (*P* < 0.001, correlation coefficient >0.70). All data presented as mean ± SD. ^#^ *P* < 0.05 vs control group, ^##^ *P* < 0.01, ^###^ *P* < 0.001 vs. control group. *P < 0.05, **P < 0.01, ***P < 0.001 vs. model group; *n* = 6–7.

In the previous experiments, the ZRH-L group performed better. Therefore, the ZRH-L group was chosen for additional studies on plasma lipidomics. UHPLC-Q-Exactive-MS/MS was used to detect plasma samples from the control, model, and ZRH-L groups. In contrast, QC samples were inserted into the sample queue every seven injections for data quality. Pearson’s correlation coefficient between QC samples was 0.99–1 (Supplementary Figure S3A), indicating that the method was stable and appropriate for these samples during the whole testing process. A total of 1,645 lipid species in ESI positive mode and 1,387 species in negative ion mode from 19 major lipid classes were identified (Supplementary Figure S3B), including glycerophosphorylcholine (PC), phosphatidylethanolamine (PE), fatty acyl (FA), triacylglycerols (TAG), phosphatidylserine (PS), phosphatidylglycerol (PG), phosphatidic acid (PA), phosphatidylinositol (PI), sphingomyelin (SM), acylcarnitine (ACar), ceramide (Cer), and glucose ceramide (HexCer). The statistical significance in specific lipid classes of plasma was calculated (Figure 5A): compared with the control group, the total content of DAG (130%), TAG (184%), Cer (131%), GlcCer (298%), CE, and SM increased significantly, while Acar (−64%), PA (−31%), PG (−29%), and PC (−36%) decreased (*P* < 0.01 or *P* < 0.05, DAG: P = 0.08) in the model group. After ZRH treatment, there was some significant downregulation of TAG (−71%), DAG (−62%), GlcCer (−13%), and upregulation of Acar (114%), PG (10%), and PA (31%) (*P* < 0.01 or *P* < 0.05, PA: *P* = 0.08) compared to the model group. The results of TAG were in agreement with lipid biochemistry.

To examine the effects of ZRH treatment on lipid metabolism in model mice, visualization of the cluster overview of all plasma samples was displayed with a PCA plot (Supplementary Figure S3C) in which the lipid compositions of the three groups significantly differed, while ZRH-L group showed separation from the model group and was close to control. The apparent separation between the model and ZRH-L groups was also observed in PLS-DA plots (Figure 5B), with high model quality (R2 of 0.84, Q2 of −0.86). According to the guidelines of VIP >1.0, FC > 1.5 or FC < 0.5, and *P* < 0.05, the volcano plot analysis revealed dramatic changes in plasma lipid composition between groups (Figures 5C,D). More than 428 species were upregulated and 850 species were downregulated in the model group compared to control. In comparison, more than 106 species were upregulated and 625 species downregulated by ZRH treatment. It is worth noting that 221 lipid species showed a significant regression after ZRH administration (Figure 5D) compared to the model group with the aforementioned guidelines and FC > 2.0 or FC < 0.5 thresholds. These 221 difference metabolites were then used to enrich and construct the probable pathways using MetaboAnalyst 5.0’s common pathway analysis feature. The glycerophospholipid metabolism was ultimately enriched with “impact values > 0.05 and -log(p) > 2.0” as a threshold (Figure 5E). Recent studies have highlighted the critical role of the glycerophospholipid metabolism in cardiovascular disease relating to oxidative stress, inflammation, endothelial dysfunction, lipid accumulation, and cardiac cell damage (Tang H, et al., 2025). The glycerophospholipid metabolism may be intimately linked to ZRH’s anti-atherosclerotic action.

Additionally, lipid markers linked to plaque vulnerability in the model group were evaluated based on VIP >1.4, FC > 5 or FC < 0.2, and Area >10^7^ thresholds. Based on this, the prospective biomarkers of ZRH’s reduced abnormal lipid were evaluated. Figure 5F depicts the variations in levels of the top-15 lipid biomarkers between the three groups. Compared to the control group, four ACars (20:4, 10:1, 16:2, (9Z)-3-hydroxyoctadecenoylcarnitine), four PEs (20:4/20:5, 20:3e/22:6, 18:4e/20:1, 1-eicosapentaenoyl-2-docosahexaenoyl-sn-glycero-3- phosphoethanolamine), two PCs (19:0/20:5, 18:1/18:2), and PS (18:2/18:2) were drastically reduced, while HexCer (d28:0/12:1) and DAG (18:0/18:0/0:0) were significantly increased in the model group. The ZRH consumption brought these substances closer to the control group (*P* < 0.05, *P* < 0.01, *P* < 0.001), while TAG levels (12:0-14:0-14:0, 12:0-14:1-16:0) were considerably lower in the ZRH group than the model group (*P* < 0.01). Therefore, these 15 lipids were identified as possible markers of lipid abnormalities decreased by ZRH. The ZRH treatment increased the amount of lipids with multiple unsaturated double bonds and decreased harmful TAG, DAG, and HexCer.

Based on the lipidomics analysis above, we examined the relationship between AS-related variables and lipidomics (Figure 5G) using *P* < 0.001 and correlation coefficient >0.70 as thresholds. The results showed that 48 lipid molecules, including 13 TAGs, seven PCs, and two SMs, were significantly correlated with AS-related factors. These 13 TAGs also showed positive correlation with the vulnerable index. In addition, seven of the 15 ZRH markers showed strong correlations with AS risk factors. Among them, TAG (12:0-14:0-14:0, 12:0-14:1-16:0) and HexCer (d28:0/12:1) had a positive correlation with the vulnerable index, and ACars (10:1), PE (20:3e/22:6) and PC (19:0/20:5) had inverse correlations. These results suggest that ZRH could stabilize plaques by regulating lipidomics.

### ZRH-IAS and active monomers regulate LPS-induced macrophage polarization

**FIGURE 6:**
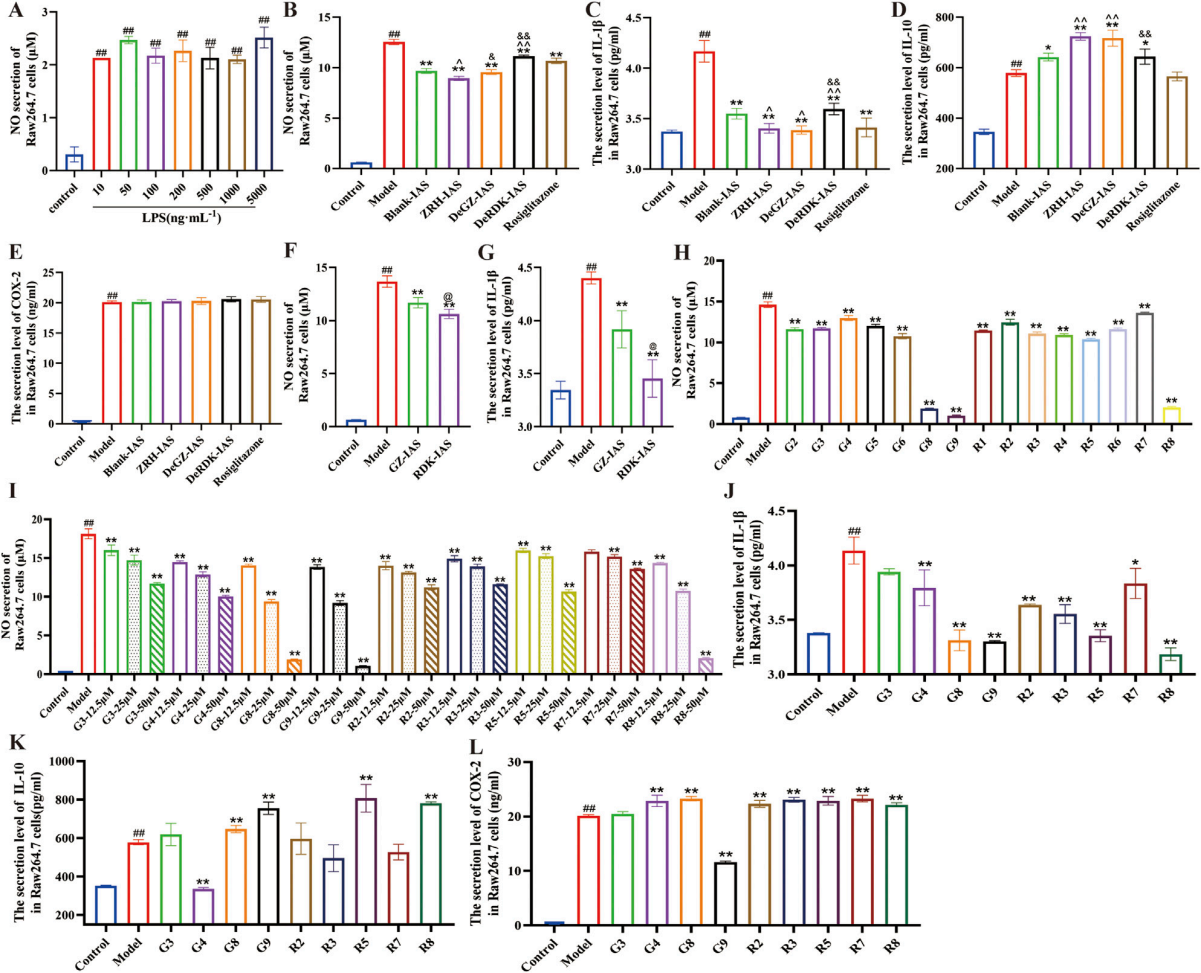
ZRH-IAS and representative metabolites shape RAW264.7 macrophage polarization. **(A)** NO concentration in RAW264.7 cells after different concentrations of LPS treatments. **(B–E)** NO concentrations, IL-1β, IL-10, and COX-2 levels in RAW264.7 cell supernatants after IAS treatments. **(F,G)** NO concentrations and IL-1β level in RAW264.7 cell supernatants after GZ-IAS or RDK-IAS treatments. **(H–L)** NO concentration and IL-1β, IL-10, and COX-2 levels in RAW264.7 cell supernatants following GZ metabolites (G3, G4, G8, G9) and RDK metabolites (R2, R3, R5, R7, R8) interventions. All data shown as mean ± SD. ^#^P < 0.05, ^##^ *P* < 0.01 vs. control group; *P < 0.05, **P < 0.01 vs. model group; ^*P* < 0.05, ^^^^*P* < 0.01 vs. Blank-IAS group; ^&^P < 0.05, ^&&^P < 0.01 vs ZRH-IAS group; ^@^ *P* < 0.01 vs. GZ-IAS group. *n* = 3 each group.

To elucidate the potential link between ZRH’s anti-atherosclerotic effects and macrophage polarization in plaque and adipose tissues, we employed an LPS-induced M1 polarization model using RAW264.7 macrophage. Model validation confirmed that treatment with 100 ng/mL LPS for 24 h successfully induced RAW264.7 macrophage M1 polarization and NO production (Figure 6A;Supplementary Figure S4). According to the CCK-8 assay, ZRH-IAS diluted above 500-fold exhibited no cytotoxicity (Zhai et al., 2024). RAW264.7 cells treated with 100 ng/mL LPS produced significantly more NO, M2-related IL-10, M1-marker IL-1β, and COX-2 than untreated cells. Intervention with ZRH-IAS, DeGZ-IAS, DeRDK-IAS, or rosiglitazone (PPARγ agonist) led to a notable decrease in NO, IL-1β, increase in IL-10, but did not affect COX-2 levels in RAW264.7 cells (Figures 6B–E). Among these, DeGZ-IAS (drug-containing IAS without GZ) outperformed DeRDK-IAS (drug-containing IAS without RDK) in inhibiting NO secretion and regulating IL-1β and IL-10 secretion. Meanwhile, the anti-inflammatory activity of RDK-IAS (RDK-containing IAS) was superior to GZ-IAS (GZ-containing IAS) (Figures 6F,G). Furthermore, seven GZ metabolites (G2, G3, G4, G5, G6, G8, and G9) and eight RDK metabolites (R1, R2, R3, R4, R5, R6, R7, and R8) (Table 1), reported for cardiovascular production (Galano et al., 2014; Godínez-Chaparro et al., 2022; Li et al., 2016; Rong et al., 2024; Liu R. et al., 2023) and abundant in ZRH pills (Bu et al., 2023), were assessed for their contribution to macrophage polarization. The findings revealed that all 15 monomers substantially lowered LPS-induced NO production at 50 μM compared with the model group (Figure 6H), among which G3, G4, G8, G9, R2, R3, R5, R7, and R8 exhibited obvious dose-dependent inhibition at 12.5–50 μM (Figure 6I). At 50 μM, G3, G4, G8, G9, R2, R3, R5, R7, and R8 significantly reduced IL-1β release (Figure 6J) and increased IL-10 secretion (Figure 6K). It is worth noting that only G9 lowered COX-2 secretion, while G4, G8, R2, R3, R5, R7, and R8 increased it (Figure 6L).

Therefore, RDK and GZ both contribute to macrophage M2 polarization and inflammatory factor secretion. However, RDK contributed more than GZ in the ZRH prescription, with the most apparent contributions coming from G8, G9, R5, and R8.

### ZRH-IAS and active monomers regulate lipid accumulation in PA-induced adipocytes

**FIGURE 7:**
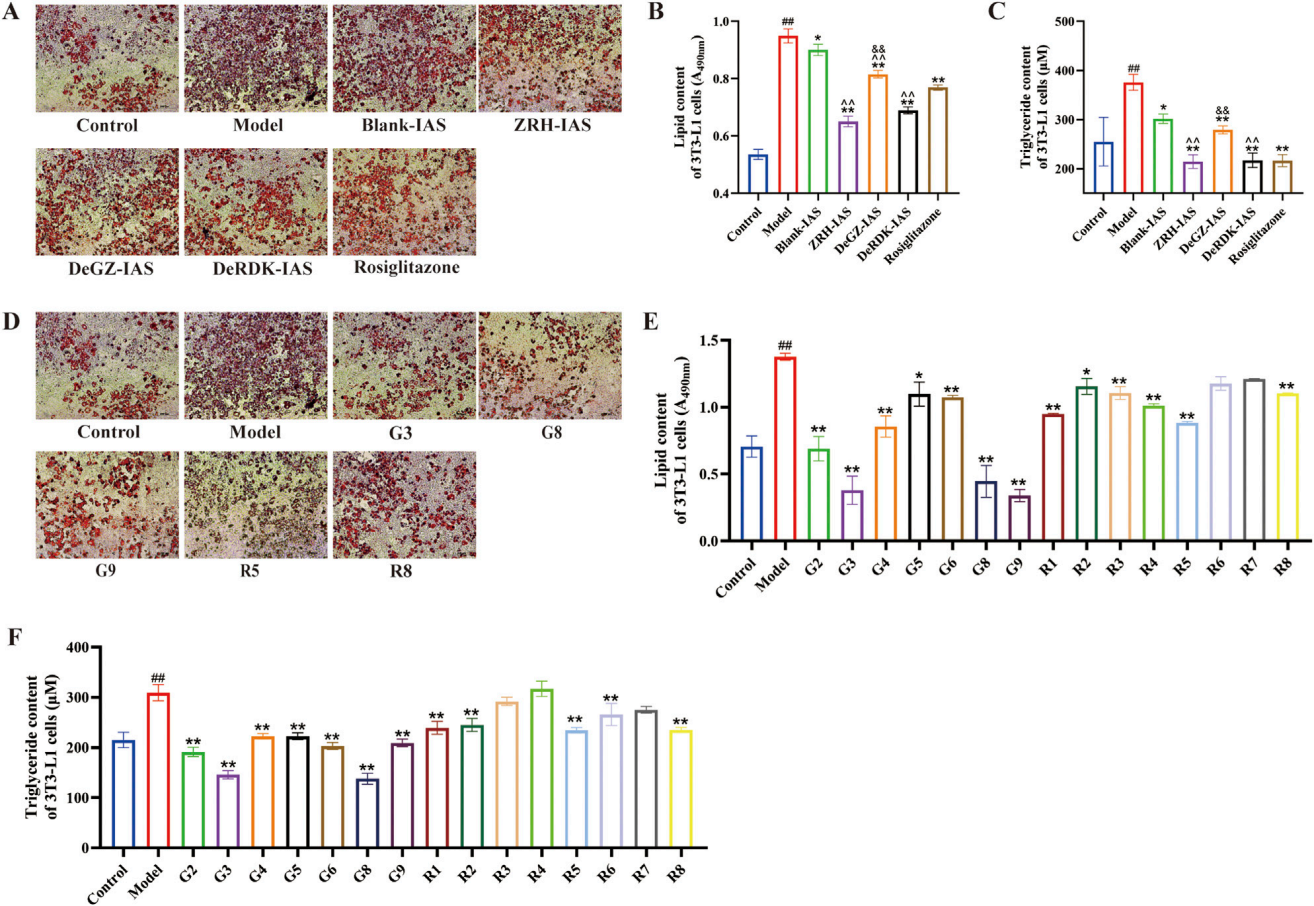
ZRH-IAS and representative metabolites inhibit adipocytes lipid deposition. **(A**,**B)** Oil red O staining and **(C)** triglyceride content in 3T3-L1 adipocytes after IAS treatments. **(D**,**E)** Oil red O staining and **(F)** triglyceride content in 3T3-L1 adipocytes following GZ metabolites (G2, G3, G4, G5, G6, G8, G9) and RDK metabolites (R1, R2, R3, R4, R5, R6, R7, R8) interventions. Scale bar = 200 μm. All data presented as mean ± SD. ^#^ *P* < 0.05, ^##^ *P* < 0.01 vs. control group; *P < 0.05, **P < 0.01 vs. model group, ^*P* < 0.05, ^^*P* < 0.01 vs Blank-IAS group; ^&^ *P* < 0.05, ^&&^ *P* < 0.01 vs ZRH-IAS group. *n* = 3 each group.

To further explore the effects of ZRH and representative metabolites on the lipid metabolism of adipose tissue, we employed a PA-induced (confluent 3T3-L1 cells) adipocyte model with abnormal lipid accumulation *in vitro*. The differentiation of 3T3-L1 preadipocytes into adipocytes was performed using a cocktail of hormones determined by Oil red O staining (Supplementary Figure S5A). When 0.2 mmol/mL PA was applied for 24 h, lipid accumulation (Supplementary Figure S5B) and triglyceride content (Supplementary Figure S5C) increased by 29% and 19%, respectively, compared to BSA (solvent for PA). In the adipocyte model, ZRH-IAS, DeGZ-IAS, DeRDK-IAS, and 10 μM-rosiglitazone intervention dramatically reversed the increased lipid deposition caused by PA (Figures 7A–C) compared to the model and Blank-IAS group (*P* < 0.01, *P* < 0.05). The ZRH-IAS group had the lowest lipid accumulation and triglyceride content, followed by the DeRDK-IAS group. Furthermore, 15 monomers were tested in the adipocyte model. The results demonstrated that 50 μmol/L G2, G3, G4, G5, G6, G8, G9, R1, R2, R5, R6, and R8 significantly reduced intracellular lipid deposition (*P* < 0.05, *P* < 0.01) and that the monomers in GZ were more effective (Figures 7D–F).

RDK and GZ thus play critical roles in regulating macrophage polarization and inhibiting aberrant lipid accumulation, each with a distinct emphasis. To investigate the synergistic effects of GZ and RDK (emperor and minister) and explore the quality markers (Q-markers) of ZRH, we developed indirect and direct co-culture models of adipocytes and macrophages. In addition, an equal mixture (MIX) of five monomers—50 μmol/L G3, G8, G9, R5, and R8—and ZRH-IAS was tested.

### ZRH-IAS and potential Q-markers (MIX) inhibit PA-induced lipid accumulation in adipocytes and LPS-induced macrophage inflammation in co-culture

**FIGURE 8:**
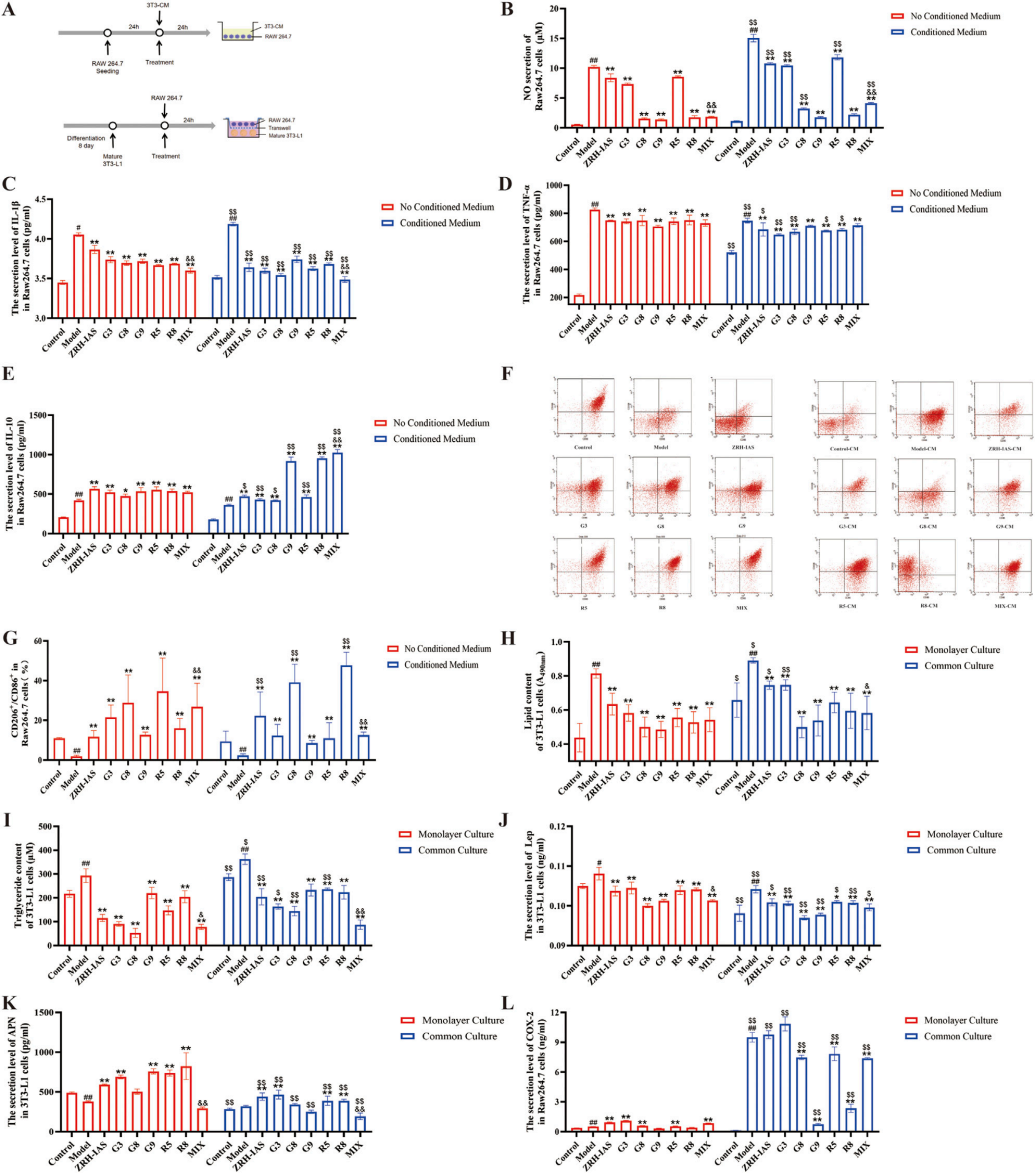
ZRH-IAS and potential Q-markers (MIX) inhibit lipid accumulation and inflammation in co-culture. **(A)** Schematic diagram of cell co-culture studies. **(B)** NO concentration and **(C–E)** level of IL-1β, TNF-α and IL-10 in the supernatant of Raw264.7 cells under indirect co-culture. **(F**,**G)** Flow cytometry evaluating M1/M2 macrophages polarization by staining CD86/CD206 under indirect co-culture. **(H)** Oil red O lipid quantification, **(I)** triglyceride content, and **(J–L)** Lep, APN, and COX-2 levels in 3T3-L1 adipocytes under direct co-culture. All data presented as mean ± SD. ^#^ *P* < 0.05, ^##^ *P* < 0.01 vs. corresponding control group at same condition; *P < 0.05, **P < 0.01 vs. model group at same condition; ^$^ *P* < 0.05, ^$$^ *P* < 0.01 vs. corresponding group without co-culture; ^&^ *P* < 0.05, ^&&^ *P* < 0.01 vs. ZRH-IAS group at same condition. *n* = 3 each group.

We used an indirect co-culture model to explore the roles of ZRH and MIX in the crosstalk between adipocyte and macrophage. Thus, after 0.2 mmol/mL PA treatment, 3T3-L1 adipocytes were treated with ZRH-IAS, G3, G8, G9, R5, R8, and MIX for 24 h, the supernatant of cells was separately collected as the 3T3-L1 adipocyte-conditioned medium (3T3/CM) to incubate the corresponding macrophage group (Figure 8A). The 3T3/CM-Model group secreted more NO, IL-1β, and TNF-α than the model group, indicating worsening inflammation (Figures 8B–D). Under indirect co-culture conditions, ZRH-IAS significantly reduced NO content, inhibited the secretion of IL-1β and TNF-α, promoted IL-10 secretion, raised the CD206^+^/CD86^+^ ratio, and enhanced the anti-inflammatory M2 phenotype transformation of macrophages. The MIX trend is consistent with ZRH-IAS, and the regulation effect is superior to or not inferior to ZRH-IAS. G8 and R8 facilitated the most anti-inflammatory M2 phenotype (Figures 8B–G).

The transwell system was then used to examine the roles of ZRH and MIX in the direct co-culture of M1 macrophages and lipid-accumulating adipocytes (Figure 8A). LPS-induced macrophages were in the upper compartment, and PA-induced 3T3-L1 adipocytes were in the lower compartment. ZRH-IAS, G3, G8, G9, R5, R8, and MIX were added separately to the lower compartment for 24 h, and the supernatant of 3T3-L1 adipocytes was analyzed. The results indicated that the macrophage proinflammatory M1 phenotypes further promoted lipid deposition on adipocytes (Figures 8H,I), as well as increased adipokine Lep and COX-2 production (Figures 8J,K). With or without the co-culture conditions, ZRH-IAS significantly inhibited adipocyte lipid accumulation and Lep secretion and promoted APN secretion. Compared to the ZRH-IAS group, MIX reduced lipid deposition, Lep, and COX-2 secretion by 57.2%,1.27% and 24.4%, respectively. However, upregulating APN secretion was weaker than ZRH-IAS. Under co-culture, G3, G8, G9, R5, and R8 significantly decreased lipid content and Lep secretion. G3, R5, and R8 promoted APN secretion, while G8, G9, R5, and R8 inhibited COX-2 secretion (Figures 8H–L).

ZRH had better anti-inflammatory and lipid-regulating properties in co-culture than in non-co-culture, indicating that its characteristics are multi-component and multi-target-organ synergistic and that MIX is an essential part of Q-markers.

### ZRH-IAS inhibits PA-induced lipid accumulation in adipocytes and LPS-induced macrophage inflammation in co-culture as a PPARγ agonist

**FIGURE 9:**
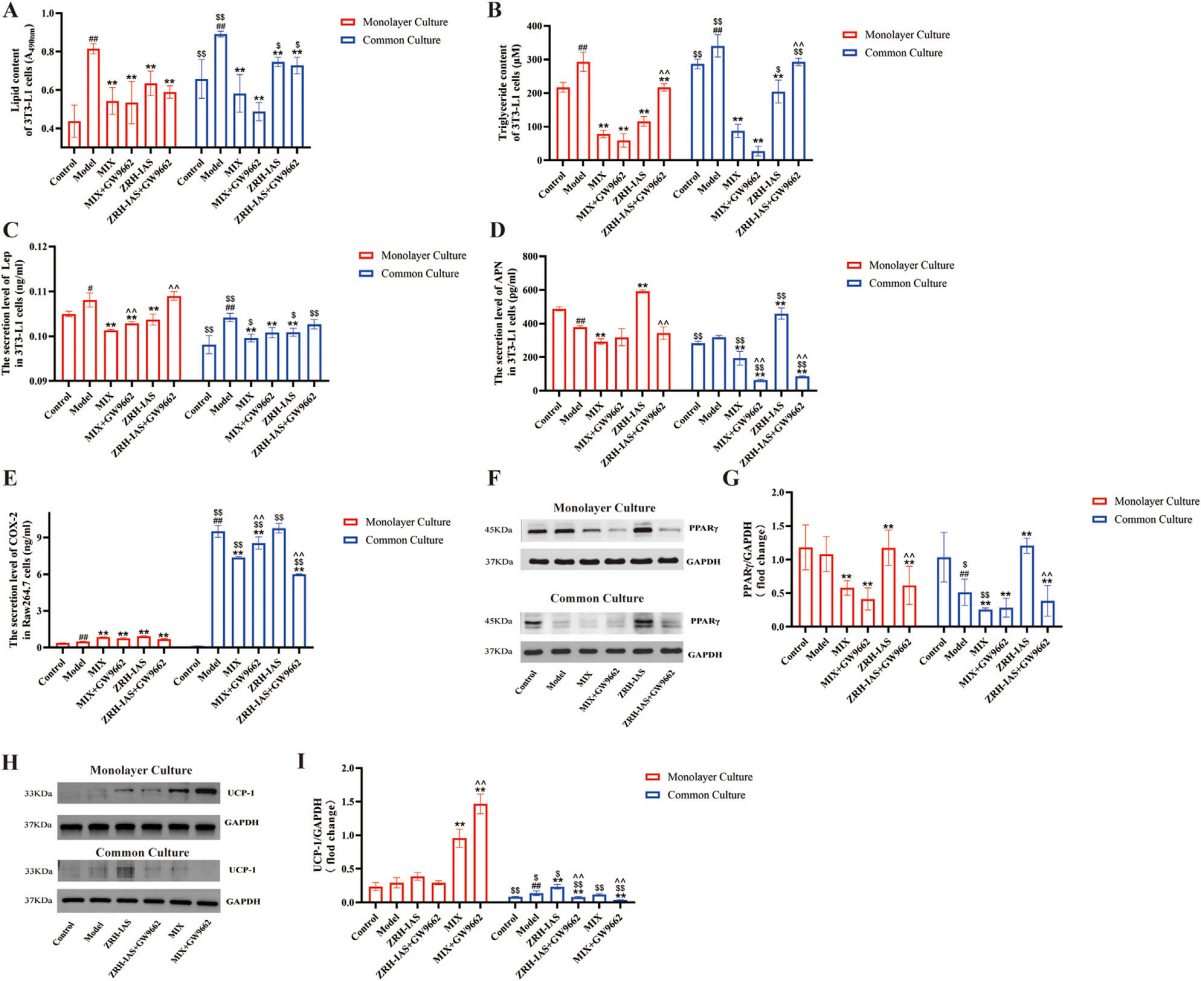
ZRH-IAS inhibited lipid accumulation and inflammation as a dual PPARγ agonist. **(A)** Oil red O lipid quantification, **(B)** triglyceride content, and level of **(C)** Lep, **(D)** APN, and **(E)** COX-2 in 3T3-L1 adipocytes with or without GW9662 treatments. Protein expression levels of **(F**,**G)** PPARγ and **(H**, **I)** UCP-1 in 3T3-L1 adipocytes with or without GW9662 treatments. All data presented as mean ± SD.^#^ *P* < 0.05, ^##^ *P* < 0.01 vs corresponding control group at same condition; *P < 0.05, **P < 0.01 vs model group at same condition; ^$^ *P* < 0.05, ^$$^ *P* < 0.01 vs corresponding group without co-culture; ^^^ *P* < 0.05, ^^^^ *P* < 0.01 vs corresponding group without GW9662 treatments. *n* = 3 each group.

Our earlier studies found that PPARγ was the co-target of many ZRH metabolites acting on CHD (Wang Q. and Zhang R, 2022; Wang et al., 2018). Accordingly, the PPAR inhibitor GW9662 was used to test whether ZRH-IAS and MIX inhibit lipid accumulation and inflammation *via* the PPAR pathway. Figure 9 shows that 10 μM GW9662 abrogated the inhibitory effect of ZRH-IAS. Thus, the ZRH-IAS + GW9662 intervention significantly increased lipid accumulation (Figures 9A,B) and Lep levels while decreasing the secretion of APN and COX-2 compared to ZRH-IAS therapy alone (Figures 9B–E). Meanwhile, there was no significant change in lipid accumulation and COX-2 levels between the MIX + GW9662 and MIX groups (Figures 9B–E). Incubating 3T3-L1 adipocytes alone resulted in no change in PPARγ expression between the model and control groups. However, in the transwell system, model adipocytes had lower PPARγ protein expression than control. The ZRH-IAS group showed an 8.6% or 134.6% rise in PPARγ expression, respectively, when incubated alone or in co-culture compared to the corresponding model group (Figures 9F,G). GW9662 abrogated PPARγ upregulation of ZRH-IAS. However, MIX did not contribute to PPARγ expression. Therefore, we further examined the expression levels of UCP-1 protein expression (Figure 9H–I). Compared to the model group, MIX and MIX + GW9662 treatment in culture alone enhanced UCP-1 protein expression (Figures 9H,I) of 3T3-L1 adipocytes by 226.1% and 400%, respectively. However, in co-culture, MIX lost UCP-1 up-regulation, which was further reduced by GW9662 (Figures 9H,I). These might explain the absence of MIX promotion on PPARγ proteins.

In summary, ZRH consists of different metabolites, and it may be a PPARγ antagonist that also activates UCP-1 expression. These metabolites work together to improve lipid metabolism and reduce inflammation. MIX may focus on modulating UCP-1, but it is only one part of ZRH’s Q-markers. Exploring the roles of different ZRH metabolites in more detail is necessary.

## Discussion

Atherosclerotic cardiovascular disease (ASCVD) remains the leading cause of mortality worldwide. The initiation of atherosclerosis fundamentally involves three processes: atherogenic lipid deposition, pro-inflammatory conditions, and endothelial dysfunction. The most common cause of myocardial infarction is ruptured atherosclerotic plaque. A large lipid core with a thin fibrous cap defines vulnerable plaque. There are a variety of factors that can promote or inhibit atherosclerotic plaque development (Nayor et al., 2021). Over the past 30 years, epidemiological studies have shown that ectopic fat deposition and metabolic abnormalities in adipose tissue contribute to atherosclerosis and cardiometabolic risk (Neeland et al., 2019). This study used short-term co-stimulation, including cold stimulation, LPS, and phenylephrine intervention, to induce atherosclerotic plaque instability in ApoE^−/−^ mice fed HFD for 18 weeks. ZRH can significantly reduce the plaque vulnerability index by decreasing lipid content and macrophage infiltration and increasing collagen content in the plaques.

High-energy intake exceeds energy expenditure, accumulating triglycerides in adipose tissue and hypertrophied adipocytes. This metabolic shift causes dyslipidemia, and proinflammatory macrophages accumulate in ectopic adipose tissue (Poledne et al., 2015). The normal function of adipose tissue is crucial to the stability of atherosclerotic plaque and the regulation of adipose tissue inflammation. PPARγ, as an important target for drug-induced adipose tissue browning, can not only improve lipid metabolism and regulate macrophage polarization state but also promote adipose tissue browning through UCP-1 (Jeon et al., 2020; Jeon et al., 2019; Wu et al., 2023). However, some studies have shown that GW9662, a PPARγ inhibitor, counteracts these beneficial effects by attenuating PPARγ agonist-mediated lipid regulation in atherosclerotic (AS) models, reversing their suppression of pro-inflammatory cytokines (IL-1β, TNF-α, IL-6) and metabolic mediators (ATGL, LPL, GLUT4), thereby exacerbating AS progression (Lee et al., 2021; Liu T. et al., 2023). In our study, GW9662 abrogated the ability of ZRH-IAS and MIX to inhibit lipid deposition and inflammation and upregulate PPARγ and UCP1 expression. Previous studies found that many factors induce “brown”, such as cold stimulation, in normal iWAT but not in mice with dysfunctional adipose tissue (Ibrahim, 2010). Similarly, *in vivo* ZRH treatment significantly reduced adipocyte hypertrophy in eWAT and BAT and increased the positive expression rate of the thermogenesis gene UCP-1 in iWAT and BAT, while ZRH-L boosted macrophage M2 polarization in PVAT. These findings collectively establish that ZRH mitigates AS by restoring the functionality of adipose tissue through the PPARγ-mediated modulation of lipid metabolism, inflammation resolution, and adipose browning.

Over the past 10 years, much research has shown that lipidomics is strongly associated with cardiovascular disease (Hemmati et al., 2023). Epidemiological findings show that increased levels of ceramides found in acute coronary syndrome (ACS) patients have been linked to insulin sensitivity and are associated with a vulnerable plaque phenotype during intravascular ultrasound. Observational and genetic epidemiological data strongly support a causal role of triglycerides (TGs) and cholesterol content within triglyceride-rich lipoproteins (TGRLs) and/or remnant cholesterol (RC) in the development of ASCVD. In addition, specific lipids, such as sphingolipid Cer, glucosylceramide (GlcCer) (Dambrova et al., 2022; Yang et al., 2022), long-chain acylcarnitines (Dambrova et al., 2022), and sphingomyelin species (Nayor et al., 2021), increase CVD risk. The plasma lipidomics showed that ZRH treatment significantly downregulates TAG, DAG, and HexCer (a subclass of GlcCer) and increases Acar, PG, and PA with multiple double bonds. Furthermore, 57 lipid molecules were significantly correlated with AS-related factors. Of them, six lipids, including TAG (12:0-14:0-14:0, 12:0-14:1-16:0), HexCer (d28:0/12:1), ACars (10:1), PE (20:3e/22:6), and PC (19:0/20:5), were recovered by ZRH. Recent research using mass spectrometry imaging revealed that the primary pathways disturbed in the aortic plaques of humans and New Zealand white rabbits were glycerophospholipid and sphingolipid metabolism (Ntshangase et al., 2025). Furthermore, hemolysis phospholipids after glycerophospholipid hydrolysis can participate in AS by activating the PPARγ pathway (Tsukahara et al., 2017; Palavicini et al., 2021). The results of differential metabolite enrichment analysis showed that ZRH could regulate glycerophospholipid metabolism and improve lipid metabolic disorders, which is consistent with the result that ZRH could upregulate PPARγ expression. The confirmation of this study’s results also provides reference value for the clinical use of lipid markers as predictors of atherosclerosis.

In the study, we explored the potential mechanisms by which ZRH alleviates AS. Adipose tissue contains many immune cells, such as macrophages and T cells (Saxton et al., 2019). Adipose tissue is a crucial endocrine organ that maintains normal physiological function by secreting adipokines and inflammatory factors. However, in chronic metabolic diseases, their secretion is dysregulated, boosting the adipokines leptin, resistin, and endolipin, as well as the inflammatory cytokines IL-6, MCP-1, and TNF-α production (Koenen et al., 2021; Liu et al., 2022); these increase plaque vulnerability in a paracrine way (Wang et al., 2023). In macrophage or adipocyte models, ZRH-IAS, DeGZ-IAS, and DeRDK-IAS significantly decreased macrophage NO and IL-1β secretion and adipocyte lipid accumulation and increased IL-10 secretion, indicating that ZRH-IAS could significantly improve macrophage inflammation and adipocyte lipid metabolism disorder. The anti-inflammatory effect of RDK is better than GZ, and GZ inhibits adipocyte accumulation more than RDK. RDK and GZ monomers contribute to efficacy, with G8, G9, R5, and R8 being the most prominent.

In addition, the monomers G3, G8, G9, R5, and R8 and their corresponding mixtures (MIX) and ZRH-IAS were tested in co-culture with macrophages and adipocytes. ZRH-IAS reduced adipocyte lipid accumulation and Lep while increasing APN and COX-2 production. MIX reduced TG, Lep, and COX-2, promoting APN secretion more than ZRH-IAS. G3, G8, and G9 reduced lipid content and inhibited Lep production, while G3, R5, and R8 increased APN secretion. Only G3 inhibited COX-2 secretion. This indicates that anti-inflammatory and anti-lipid accumulation is a multi-component synergistic effect of ZRH. MIX is a representative composition of ZRH, which may be an essential part of the Q-markers group of ZRH.

PPARs are essential regulators of metabolism and inflammation in adipose tissue. PPARγ orchestrates UCP1 transcription during brown adipocyte differentiation. By promoting the storage of fatty acids and inhibiting their breakdown, PPARγ helps maintain energy balance in the body (Choi, S.S., et al., 2016; Lamichane et al., 2018; Kroon et al., 2020). In addition to its role in metabolic diseases, PPARγ also plays a role in inflammation and immune responses (Ridder and Schwaninger, 2012; Lin et al., 2010). In this study, ZRH-L treatment boosted PPARγ expression in BAT and eWAT of ApoE^−/−^ model mice. *In vitro*, GW9662 (a PPARγ inhibitor) reversed the effect of ZRH-IS on lipid content, Lep, APN, and COX-2 secretion levels and protein expression of UCP-1 and PPARγ in adipocytes. However, the MIX + GW9662 group had no significant difference in TG or COX-2 level or in PPARγ expression compared with the MIX group. In summary, the regulation of PPARγ is an essential mechanism for inhibiting lipid metabolism and the anti-inflammatory effects of ZRH; MIX may focus on modulating UCP-1—it is only one part of ZRH’s Q-markers. Exploring the roles of different ZRH metabolites in more detail is necessary.

In conclusion, AS is a chronic inflammatory disease driven by a disorder of the lipid metabolism. In this study, we found that ZRH as a PPARγ activating medication can regulate inflammatory factors and adipokines, suppress lipid metabolism disorders and inflammation, enhance adipose tissue “brown,” increase AS plaque stability, and improve plasma lipidomic features. This study provides an essential experimental basis for treating atherosclerosis with ZRH, investigating its compatibility mechanism and the potential quality markers of ZRH.

## Data Availability

The original contributions presented in the study are publicly available. This data can be found here: https://figshare.com/articles/dataset/Analysis_of_Lipidomics_of_Zhuriheng_Pills/30330013, doi: 10.6084/m9.figshare.30330013.
