# Correction: Zhuriheng pills improve adipose tissue dysfunction and inflammation by modulating PPARγ to stabilize atherosclerotic plaques

**DOI:** 10.3389/fphar.2025.1736989

**Published:** 2025-11-13

**Authors:** Qiong Zhai, Fangyuan Liang, Guanlin Yang, Zhimin Liu, Xin Dong, Ren Bu, Peifeng Xue, Shengsang Na, Xuan Zhang, Pengwei Zhao, Xiaoning Wang, Qiang Wei, Yuewu Wang, Jingkun Lu

**Affiliations:** 1 School of Basic Medicine, Inner Mongolia Medical University, Huhehot, China; 2 School of Pharmaceutical and Environmental Engineering, Nantong Vocational University, Nantong, China; 3 Pharmaceutical Department, Zaozhuang Thoracic hospital (Zaozhuang Cancer Hospital), Zaozhuang, China; 4 Pharmaceutical Department, Chifeng College Affiliated Hospital, Chifeng, China; 5 School of Pharmacy, Inner Mongolia Medical University, Huhehot, China; 6 Medical Innovation Center for Nationalities, Inner Mongolia Medical University, Huhehot, China

**Keywords:** Zhuriheng pills, atherosclerotic plaques, the adipose tissue dysfunction and inflammation, PPARγ signaling pathway, the adipose tissue dysfunction, the adipose tissue inflammation

The order of authors in the **Author list** of the published paper was erroneously given as:

“Qiong Zhai1†, Fangyuan Liang2†, Guanlin Yang3†, Zhimin Liu4, Xin Dong5, Ren Bu5, Peifeng Xue5, Shengsang Na6, Xuan Zhang1, Pengwei Zhao1, Xiaoning Wang1, Qiang Wei1, Jingkun Lu1* and Yuewu Wang5*”

The correct **Author list** reads:

“Qiong Zhai1†, Fangyuan Liang2†, Guanlin Yang3†, Zhimin Liu4, Xin Dong5, Ren Bu5, Peifeng Xue5, Shengsang Na6, Xuan Zhang1, Pengwei Zhao1, Xiaoning Wang1, Qiang Wei1, Yuewu Wang5* and Jingkun Lu1*”

The original article has been updated.

