# Supplementary material for: Zhuriheng pills improve adipose tissue dysfunction and inflammation by modulating PPARγ to stabilize atherosclerotic plaques
Source: Front Pharmacol. 2025 Oct 20;16:1576521. doi: 10.3389/fphar.2025.1576521 (PMC12580357; doi:10.3389/fphar.2025.1576521)
Supplement: Supplementary file 1 [file Supplementaryfile1.docx]

**Method validation**

Validation methods were carried out by the Chinese Pharmacopoeia (2020 Edition, Part IV 9012) Guidance on Bioanalytical Method Validation. The lower limit of quantitation (LLOQ) for each standard was determined using the curve's lowest point.

***Specificity***

As shown in Fig.S1, it was found that citric acid (III) Interference peaks were detected in blank intestinal solution in rats ZRH-IAS, but only 20% of what was detected in LLOQ samples, with no significant interference peaks detected in the corresponding retention times for other analytes.

**
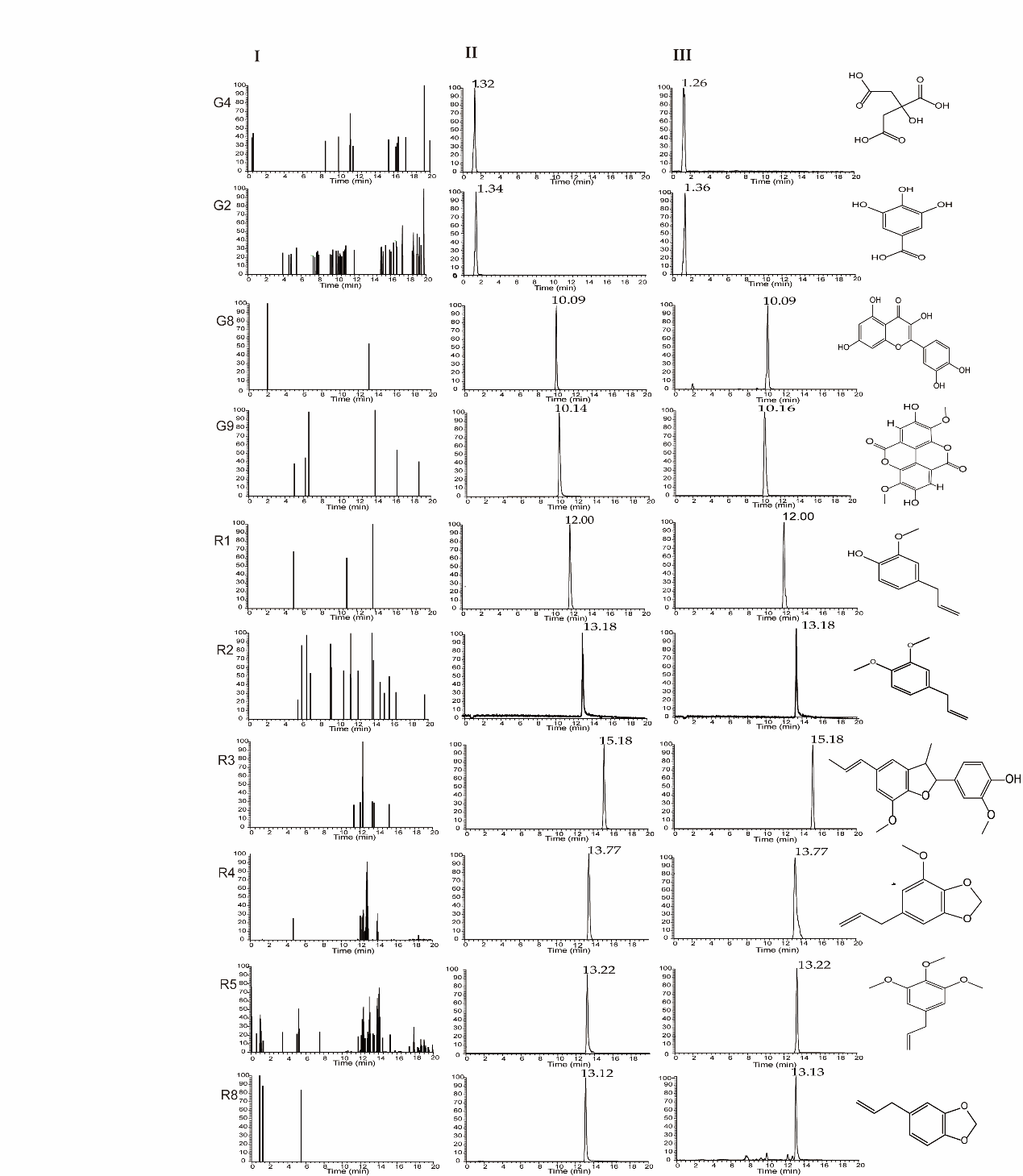
**

**Fig.S1** Extracted-ion chromatograms of 10 analytes in (I) Blank-IAS, (II) QC at the middle quality control, (III) ZRH15-IAS samples. The analytes include gallic acid (G2), citric acid (G4), quercetin (G8), and 3,3'-Di-O-methylellagic acid (G9) in negative ion mode, and eugenol (R1), methyleugenol (R2), dehydrodiisoeugenol (R3), myristicin (R4), elemicin (R5), and safrole (R8) in positive ion mode. Blank-IAS: blank intestinal absorbed solution, ZRH15-IAS: drug-containing intestinal absorbed sample collected after 37.8 mg·mL^-1^ ZRH treatment for 2 hrs, QC: blank intestinal absorbed sample spiked with standards.

***Linearity and LLOQ***

The linear equations, linear ranges, correlation coefficients, and LLOQ for 10 analytes in IAS were presented in Table S1. The calibration curves showed good linearity at concentration ranges of 10 analytes with the coefficient of determination (*r^2^* ) value greater than 0.995 for all analytes. The accuracy (RE, %) of 10 analytes ranged from -2.68% and 4.59%, and RSD < 3.5%. Therefore, this showed that the HPLC-Q-Exactive-MS/MS method was sensitive and appropriate for quantifying these analytes in IAS samples.

**Table S1.** The regression equations, linear ranges, and LLOQ of 10 analytes in IAS quality control (QC) samples (*n=*6).

| **Analytes** | **Linear ranges** | | | **LLOQ** | | |
| --- | --- | --- | --- | --- | --- | --- |
|  | **Linear equation^a^** | **Correlation coefficient**  **(*γ^2^*)** | **Linear range**  **(ng/ml)** | **Measured Conc. (ng/ml)** | **Accuracy**  **(RE, %)** | **Precision（RSD, %）** |
| Gallic acid (G2) | *y* = 0.0046*x* - 0.04 | 0.9957 | 100.0-100000.0 | 99.99±1.48 | -0.01 | 1.48 |
| Citric acid (G4I) | *y* = 0.0018*x* + 0.2467 | 0.9969 | 500.0-500000.0 | 522.9±8.05 | 4.59 | 3.08 |
| Quercetin (G8) | y = 0.0206*x* + 0.00012 | 0.9969 | 0.5-500.0 | 0.49±0.01 | -2.68 | 1.30 |
| 3,3'-Di-O-dimethyl ellagic acid (G9) | y = 0.0067*x* + 0.0179 | 0.9997 | 500.0-500000.0 | 519.8±6.71 | 3.93 | 1.29 |
| Eugenol (R1) | y = 0.0008*x* + 0.0058 | 0.9985 | 100.0-100000.0 | 102.35±1.60 | 2.35 | 1.62 |
| Methyl eugenol (R2) | y = 0.0017*x* + 0.0012 | 0.9955 | 0.5-500.0 | 0.50±0.01 | 0.14 | 0.40 |
| Dehydrodiisoeugenol (R3) | y = 0.0716*x* + 0.0503 | 0.9993 | 100.0-100000.0 | 101.44±15.84 | 1.44 | 0.49 |
| Myristicin (R4) | y = 0.0002*x*+0.0061 | 0.9958 | 20.0-20000.0 | 20.03±0.69 | 0.13 | 3.43 |
| Elemicin (R5) | y = 0.0168*x* + 0.0051 | 0.9988 | 50.0-50000.0 | 50.67±1.46 | 1.34 | 2.88 |
| Safrole (R8) | y = 0.0008x + 0.1287 | 0.9965 | 50.0-50000.0 | 50.28±0.72 | 0.56 | 1.45 |
| Node: “a”, y: concentration, x: peak area ratio. IAS: intestinal absorbed solution. | | | | | | |

***Precision and accuracy***

The intra- and inter-day precision and accuracy values for QC samples at three different concentrations are shown in Table S2 with precision (RSD <8.80%) and accuracy (RE, -6.19%~7.53%). These results confirm that this method is sufficiently precise and accurate.

**Table S2.** Precision and accuracy of 10 analytes in rat intestinal solution quality control (QC) samples (*n*=6).

| **Analytes** | **Spiked Conc. (ng/ml)** | **Intra-day** | | **Inter-day** | | **Analytes** | **Spiked Conc. (ng/ml)** | **Intra-day** | | **Inter-day** | |
| --- | --- | --- | --- | --- | --- | --- | --- | --- | --- | --- | --- |
|  |  | **Precision(RSD%)** | **Accuracy (RE, %)** | **Precision**  **(RSD%)** | **Accuracy (RE, %)** |  |  | **Precision(RSD%)** | **Accuracy (RE, %)** | **Precision**  **(RSD%)** | **Accuracy (RE, %)** |
| Gallic acid(G2) | 80000 | 1.90 | 2.07 | 0.99 | -1.2 | Methyl eugenol(R2) | 400 | 3.10 | 6.02 | 5.86 | 2.87 |
|  | 4000 | 2.43 | 6.07 | 2.93 | 0.18 |  | 20 | 3.90 | 7.53 | 8.80 | -0.97 |
|  | 200 | 3.33 | 5.55 | 3.81 | -4.72 |  | 1 | 1.64 | 6.03 | 3.74 | 0.07 |
| Citric acid(G4) | 400000 | 2.58 | -1.62 | 5.14 | 0.06 | Dehydrodiisoeugenol(R3) | 80000 | 1.22 | -0.57 | 4.01 | 1.53 |
|  | 20000 | 1.55 | -0.74 | 2.80 | -1.98 |  | 4000 | 2.69 | 4.71 | 2.95 | -4.09 |
|  | 1000 | 2.22 | -4.42 | 3.71 | 2.42 |  | 200 | 1.99 | -4.59 | 5.86 | 2.87 |
| Quercetin(G8) | 400 | 1.31 | 1.91 | 2.36 | 0.22 | Myristicin (R4) | 16000 | 0.63 | 4.78 | 3.33 | 1.7 |
|  | 20 | 4.83 | 7.24 | 5.07 | 5.14 |  | 800 | 0.92 | 4.36 | 3.36 | 1.32 |
|  | 1 | 3.41 | -4.98 | 6.54 | 0.58 |  | 40 | 1.33 | 5.74 | 5.50 | 1.02 |
| 3,3'-Di-O-dimethyl ellagic acid(G9) | 400000 | 1.25 | -1.37 | 2.09 | 0.29 | Elemicin(R5) | 40000 | 1.23 | 0.11 | 1.57 | -0.91 |
|  | 20000 | 2.81 | 3.61 | 2.01 | 3.93 |  | 2000 | 1.74 | 4.29 | 4.57 | 0.44 |
|  | 1000 | 1.32 | -6.19 | 1.64 | -2.07 |  | 100 | 1.42 | 2.37 | 3.91 | -0.5 |
| Eugenol(R1) | 80000 | 1.49 | 4.33 | 1.94 | 2.81 | Safrole(R8) | 40000 | 6.95 | -0.72 | 5.10 | -0.6 |
|  | 4000 | 3.65 | 6.83 | 3.07 | 5.94 |  | 2000 | 3.49 | -2.27 | 2.81 | -0.92 |
|  | 200 | 2.99 | 7.36 | 3.85 | 6.65 |  | 100 | 2.19 | -1.46 | 4.22 | 2.53 |

***Recovery and matrix effect***

The recovery and matrix effect of 10 analytes in IAS were evaluated by extracting with acidified acetonitrile and methanol/the methanol precipitation method. As shown in Table S3, the extraction recovery and the matrix effect were observed for 10 analytes in IAS samples within the testing concentration range. The results indicated a higher extraction recovery (≥91%) and fewer matrix effects (94.53%～106.80%). Sample stability was not affected by the preparation procedure.

**Table S3.** Recovery and matrix effects of 10 analytes and digoxin (IS) in rat intestinal solution quality control (QC) sample (*n*=6).

| **Analytes** | **Spiked Conc. (ng/ml)** | **Recovery^a^**  **(%, mean±SD)** | **Matrix effect^b^ (%, mean±SD)** | **Analytes** | **Spiked Conc. (ng/ml)** | **Recovery^a^**  **(%, mean±SD)** | **Matrix effect^b^(%, mean±SD)** |
| --- | --- | --- | --- | --- | --- | --- | --- |
| Gallic acid(G2) | 80000 | 95.21±1.42 | 102.24±0.68 | Methyl eugenol(R2) | 400 | 93.46±6.40 | 95.50±2.75 |
|  | 4000 | 105.83±3.72 | 106.76±1.09 |  | 20 | 95.65±3.36 | 98.57±1.67 |
|  | 200 | 100.26±4.07 | 105.8±0.97 |  | 1 | 91.61±3.66 | 97.23±0.67 |
| Citric acid(G4) | 400000 | 96.07±1.37 | 105.70±2.72 | Dehydrodiisoeugenol(R3) | 80000 | 99.85±6.66 | 97.78±1.29 |
|  | 20000 | 99.91±2.10 | 106.68±1.67 |  | 4000 | 98.47±5.14 | 100.14±1.31 |
|  | 1000 | 95.27±2.59 | 110.06±1.82 |  | 200 | 96.57±1.29 | 96.84±0.40 |
| Quercetin(G8) | 400 | 94.70±0.54 | 94.81±0.73 | Myristicin (R4) | 16000 | 105.14±1.18 | 95.87±3.62 |
|  | 20 | 93.49±0.30 | 95.91±0.19 |  | 800 | 91.20±3.09 | 104.77±2.01 |
|  | 1 | 103.97±1.71 | 96.95±0.16 |  | 40 | 94.51±1.66 | 96.34±1.52 |
| 3,3'-Di-O-dimethyl ellagic acid(G9) | 400000 | 99.71±0.85 | 94.53±2.54 | Elemicin(R5) | 40000 | 98.66±3.92 | 103.00±0.86 |
|  | 20000 | 95.52±1.79 | 96.59±3.04 |  | 2000 | 96.15±1.72 | 103.69±1.17 |
|  | 1000 | 93.14±3.92 | 99.42±3.26 |  | 100 | 96.89±5.75 | 103.77±0.97 |
| Eugenol(R1) | 80000 | 101.12±3.107 | 96.38±2.40 | Safrole(R8) | 40000 | 98.65±1.43 | 103.46±1.49 |
|  | 4000 | 97.79±2.82 | 98.10±3.84 |  | 2000 | 105.58±0.91 | 106.80±2.92 |
|  | 200 | 99.63±6.30 | 96.36±2.31 |  | 100 | 105.17±2.47 | 98.03±3.68 |
| Node： “a”，Matrix effect is expressed as the ratio of the mean peak area of an analyte spiked post-extraction to the mean peak area of the same analyte standards multiplied by 100. “b”， Recovery is calculated as the ratio of the mean peak area of an analyte–spiked intestinal solution before extraction to the mean peak of an analyte spiked after extraction of blank intestinal solution multiplied by 100. | | | | | | | |

***Stability***

Table S4 shows the stability evaluations for long-term storage, short-term storage, and post-preparation storage. The QC samples prepared from rat IAS showed no significant degradation under the different conditions, with an RSD of less than 10%, which indicated that the biological samples met the analytical requirements. Therefore, IAS samples should be processed within 2 hrs at room temperature or 4 wks at −80°C and injected into the HPLC-Q-Exactive-MS/MS system within 12 hrs of preparation.

**Table S4.** Stability of 10 analytes in rat intestinal solution quality control (QC) samples (*n*=6).

| **Analytes** | | **Spiked Conc.**  **(ng/ml)** | **post-preparation stability** | | **Short-term stability** | | **Long-term stability** | |  |  |  |
| --- | --- | --- | --- | --- | --- | --- | --- | --- | --- | --- | --- |
|  |  |  | **Measured Conc. (ng/ml)** | **RSD (%)** | **Measured Conc.**  **(ng/ml)** | **RSD (%)** | **Measured Conc.**  **(ng/ml)** | **RSD (%)** |  |  |  |
| Gallic acid (G2) | | 80000 | 80152.39±3831.27 | 4.78 | 83608.55±2809.23 | 3.36 | 82688.51±2505.45 | 3.03 |  |  |  |
|  |  | 4000 | 4098.84±234.04 | 5.71 | 3900.46±40.17 | 1.03 | 4142.47±185.17 | 4.47 |  |  |  |
|  |  | 200 | 220.04±7.57 | 3.44 | 197.94±4.49 | 2.27 | 202.28±15.72 | 7.77 |  |  |  |
| Citric acid (G4) | | 400000 | 410564.5±15437.1 | 3.76 | 421245.8±10446.8 | 2.48 | 416683.8±5416.8 | 1.30 |  |  |  |
|  |  | 20000 | 203708.2±1279.7 | 6.28 | 20802.9±1297.9 | 6.24 | 20338±498.3 | 2.45 |  |  |  |
|  |  | 1000 | 1018.6±45.42 | 4.46 | 1071.9±27.4 | 2.56 | 1026.3±62.4 | 6.08 |  |  |  |
| Quercetin(G8) | 400 | 410.52±12.89 | 3.14 | 382.52±7.65 | 2.00 | 392.42±20.64 | 5.26 |  |  |  |  |
|  | 20 | 19.228±0.49 | 2.55 | 21.022±1.07 | 5.09 | 19.82±1.01 | 5.10 |  |  |  |  |
|  | 1 | 0.99±0.023 | 2.27 | 1.093±0.02 | 1.73 | 0.98±0.09 | 8.98 |  |  |  |  |
| 3,3'-Di-O-dimethyl ellagic acid(G9) | 400000 | 420200.28±11891.66 | 2.83 | 413280.34±7687.01 | 1.86 | 396440.24±14271.84 | 3.60 |  |  |  |  |
|  | 20000 | 19770.43±290.62 | 1.47 | 19998.165±757.925 | 3.79 | 20424±927.25 | 4.54 |  |  |  |  |
|  | 1000 | 1034.64±52.04 | 5.03 | 1031.63±17.02 | 1.65 | 980.4±38.825 | 3.96 |  |  |  |  |
| Eugenol(XIX) | | | | 80000 | 77136.98±2314.08 | 3.00 | 81224.26±7432.12 | 9.15 | 79048.92±5968.12 | 7.55 |  |
|  |  |  |  | 4000 | 3822.86±95.19 | 2.49 | 3974.19±114.45 | 2.88 | 4053.65±324.69 | 8.01 |  |
|  |  |  |  | 200 | 192.27±6.38 | 3.32 | 201.44±8.68 | 4.31 | 202.48±11.46 | 5.66 |  |
| Methyl eugenol(XXII) | | | 400 | 389.24±25.11 | 6.45 | 397.88±23.87 | 6.00 | 385.52±24.40 | 6.33 |  |  |
|  |  |  | 20 | 20.38±1.21 | 5.92 | 20.33±1.23 | 6.03 | 20.33±1.81 | 8.88 |  |  |
|  |  |  | 1 | 1..00±0.034 | 3.39 | 1.02±0.03 | 3.23 | 1.02±0.09 | 8.95 |  |  |
|  | |  |  |  |  |  |  |  |  |  |  |
|  |  |  |  |  |  |  |  |  |  |  |  |
|  |  |  |  |  |  |  |  |  |  |  |  |
| Dehydrodiisoeugenol(XXIV) | | 80000 | 78624.65±1328.75 | 1.69 | 81728±997.08 | 1.22 | 79344.53±5681.03 | 7.16 |  |  |  |
|  |  | 4000 | 3881.61±210.77 | 5.43 | 3970±73.05 | 1.84 | 4116.19±100.02 | 2.43 |  |  |  |
|  |  | 200 | 189.04±2.87 | 1.52 | 201.68±3.00 | 1.49 | 207.24±10.67 | 5.15 |  |  |  |
| Myristicin (XVIII) | | 16000 | 15716.83±721.40 | 4.59 | 15793.65±176.89 | 1.12 | 15713.69±982.1 | 6.25 |  |  |  |
|  |  | 800 | 766.64±14.26 | 1.86 | 788.08±2.88 | 3.03 | 788.24±39.81 | 5.05 |  |  |  |
|  |  | 40 | 39.38±3.50 | 8.90 | 40.012±2.12 | 5.31 | 37.81±1.66 | 4.39 |  |  |  |
| Elemicin(XXI) | | 40000 | 40632.96±2819.86 | 6.94 | 39060.25±3534.93 | 9.05 | 40192.53±2290.94 | 5.70 |  |  |  |
|  |  | 2000 | 1986.28±120.96 | 6.09 | 2038.2±43.41 | 2.13 | 1979.27±115.58 | 5.84 |  |  |  |
|  |  | 100 | 102.62±5.46 | 5.32 | 102.76±3.72 | 3.62 | 98.61±7.45 | 7.55 |  |  |  |
| Safrole(R8) | | | | | 40000 | 39620.91±1727.43 | 4.36 | 40400±1050.4 | 2.60 | 40324.72±1028.26 | 2.55 |
|  |  |  |  |  | 2000 | 2052.81±32.64 | 1.59 | 1932.61±120.21 | 6.22 | 2017±51.03 | 2.53 |
|  |  |  |  |  | 100 | 103.27±4.39 | 4.25 | 99.88±2.60 | 2.60 | 104.73±6.26 | 5.98 |
