# Supplementary material for: Zhuriheng pills improve adipose tissue dysfunction and inflammation by modulating PPARγ to stabilize atherosclerotic plaques
Source: Front Pharmacol. 2025 Oct 20;16:1576521. doi: 10.3389/fphar.2025.1576521 (PMC12580357; doi:10.3389/fphar.2025.1576521)
Supplement: Supplementary file 2 [file Supplementaryfile5.docx]

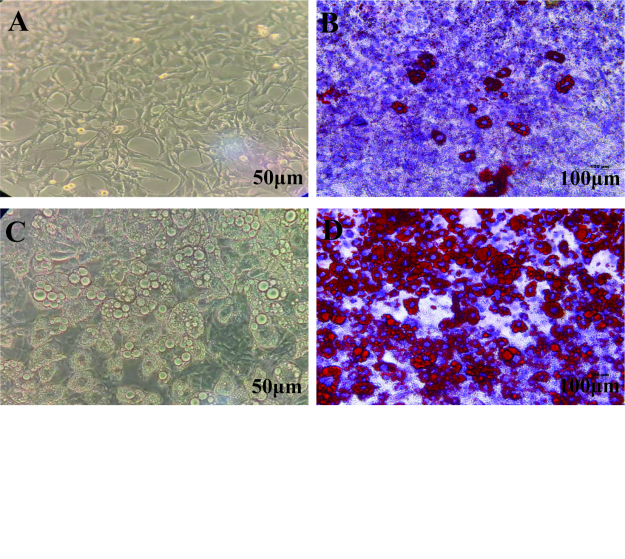


**FigS5A** Differentiation plots of the 3T3-L1 preadipocytes. (A, B) no Oil red staining and Oil red staining of the uninduced 3T3-L1 adipocytes; (C, D) no Oil red staining and Oil red staining of the 3T3-L1 adipocytes after drug induction.


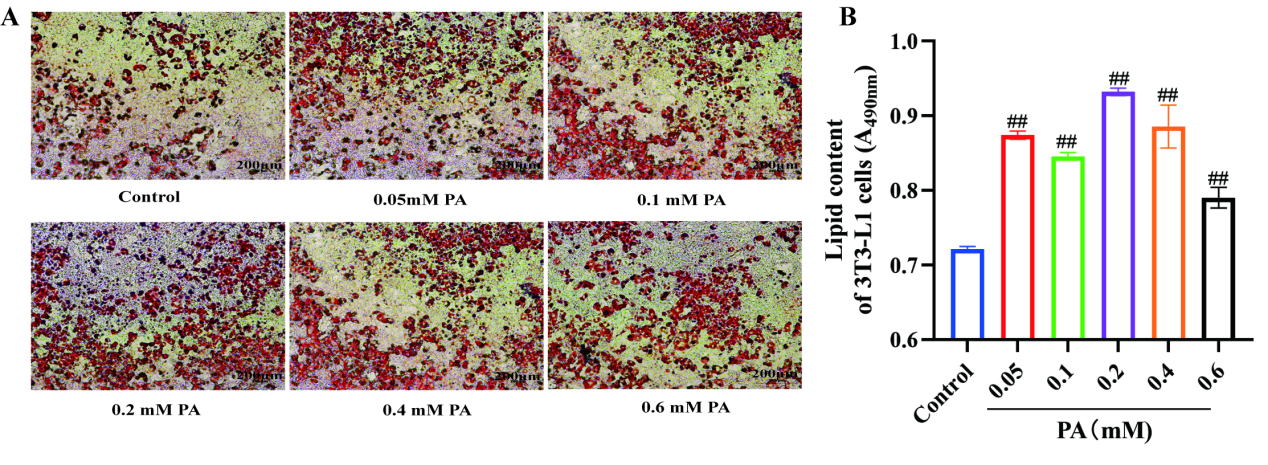


**FigS5B** Oil red O staining and Lipid content in 3T3-L1 adipocytes after different concentration PA treatments. All data are shown as mean ± SD. ^#^P < 0.05, ^##^*P* < 0.01 *vs.* control group.


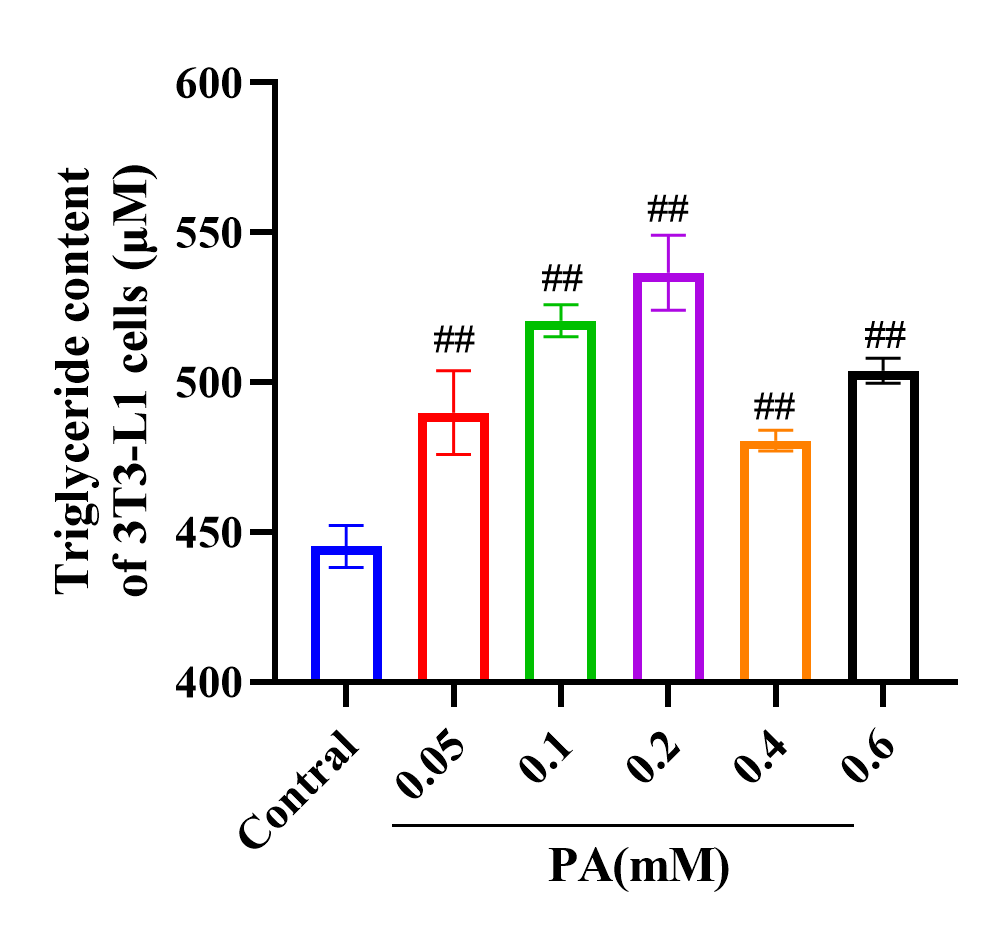


**FigS5C** Triglyceride content in 3T3-L1 adipocytes after different concentration PA treatments. All data are shown as mean ± SD. ^#^P < 0.05, ^##^*P* < 0.01 *vs.* control group.
