# Supplementary material for: Zhuriheng pills improve adipose tissue dysfunction and inflammation by modulating PPARγ to stabilize atherosclerotic plaques
Source: Front Pharmacol. 2025 Oct 20;16:1576521. doi: 10.3389/fphar.2025.1576521 (PMC12580357; doi:10.3389/fphar.2025.1576521)
Supplement: Supplementary file 3 [file Supplementaryfile3.docx]

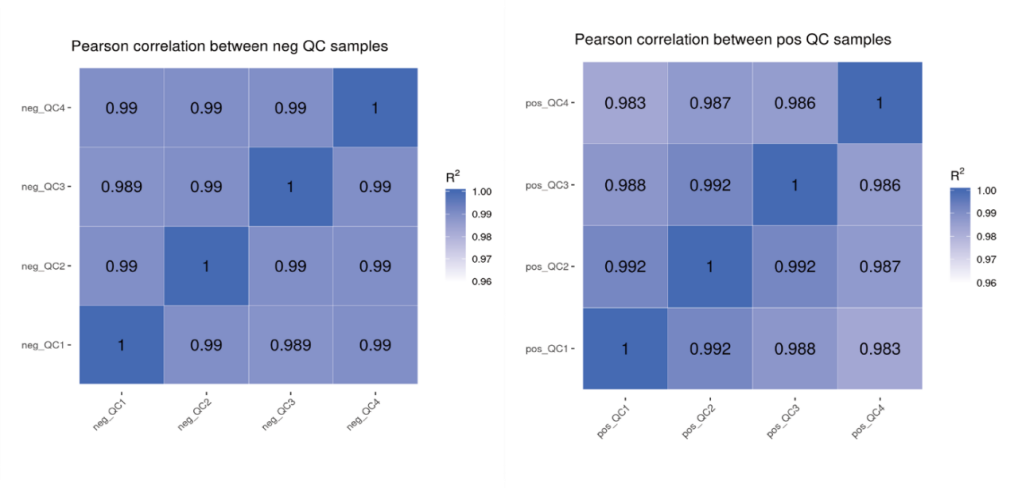


Fig.S3A Pearson correlation coefficients between plasma QC samples

Note: Left - negative ion mode; right - positive ion mode.


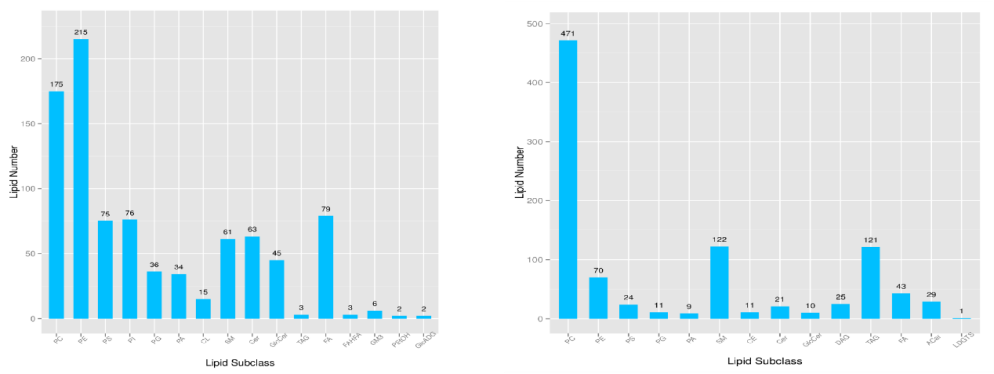


Fig.S3B Lipid subclass analysis

Note: Vertical axis represents the number of lipid subclasses containing lipid compounds, horizontal axis represents the lipid subclass. Left - negative ion pattern; Right - positive ion pattern.


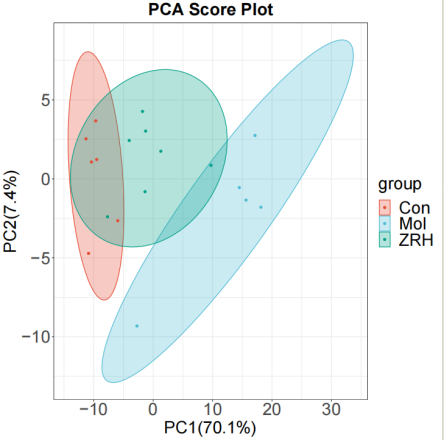


Fig.S3C PCA plots of the blood lipid samples
