# Supplementary material for: Zhuriheng pills improve adipose tissue dysfunction and inflammation by modulating PPARγ to stabilize atherosclerotic plaques
Source: Front Pharmacol. 2025 Oct 20;16:1576521. doi: 10.3389/fphar.2025.1576521 (PMC12580357; doi:10.3389/fphar.2025.1576521)
Supplement: Supplementary file 4 [file Supplementaryfile2.docx]

In the fourth week, mice fed HFD had significantly higher body weight than mice fed a normal diet (Fig.S2A). At the end of test, the mass index of eWAT, BAT and eWAT to body weight (g/g) and body weight in Model mice were no significant variations, or the mass index of adipose tissue (g/g) between Model and the drug interventions groups (Fig. S2B-F).


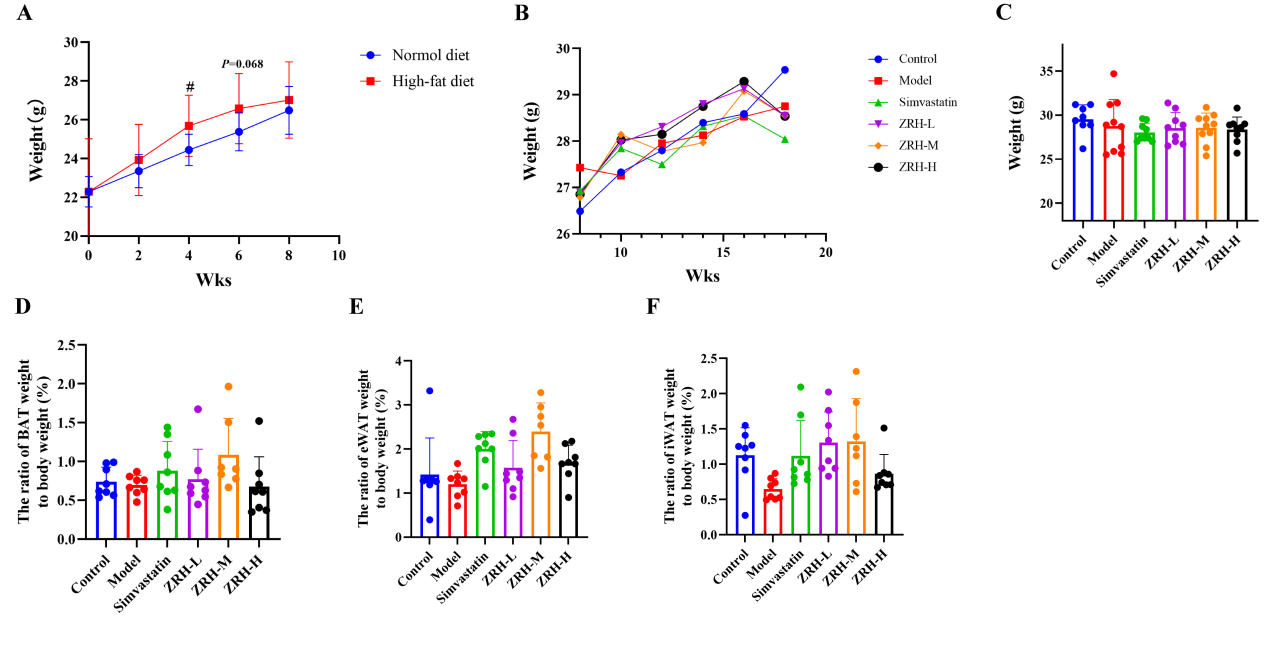


All the data are presented as mean ± SD. ^#^*P* < 0.05, ^##^*P* < 0.01 *vs.* control group; **P* < 0.05, ***P* < 0.01 *vs.* model group.
