# Supplementary material for: Zhuriheng pills improve adipose tissue dysfunction and inflammation by modulating PPARγ to stabilize atherosclerotic plaques
Source: Front Pharmacol. 2025 Oct 20;16:1576521. doi: 10.3389/fphar.2025.1576521 (PMC12580357; doi:10.3389/fphar.2025.1576521)
Supplement: Supplementary file 5 [file Supplementaryfile4.docx]

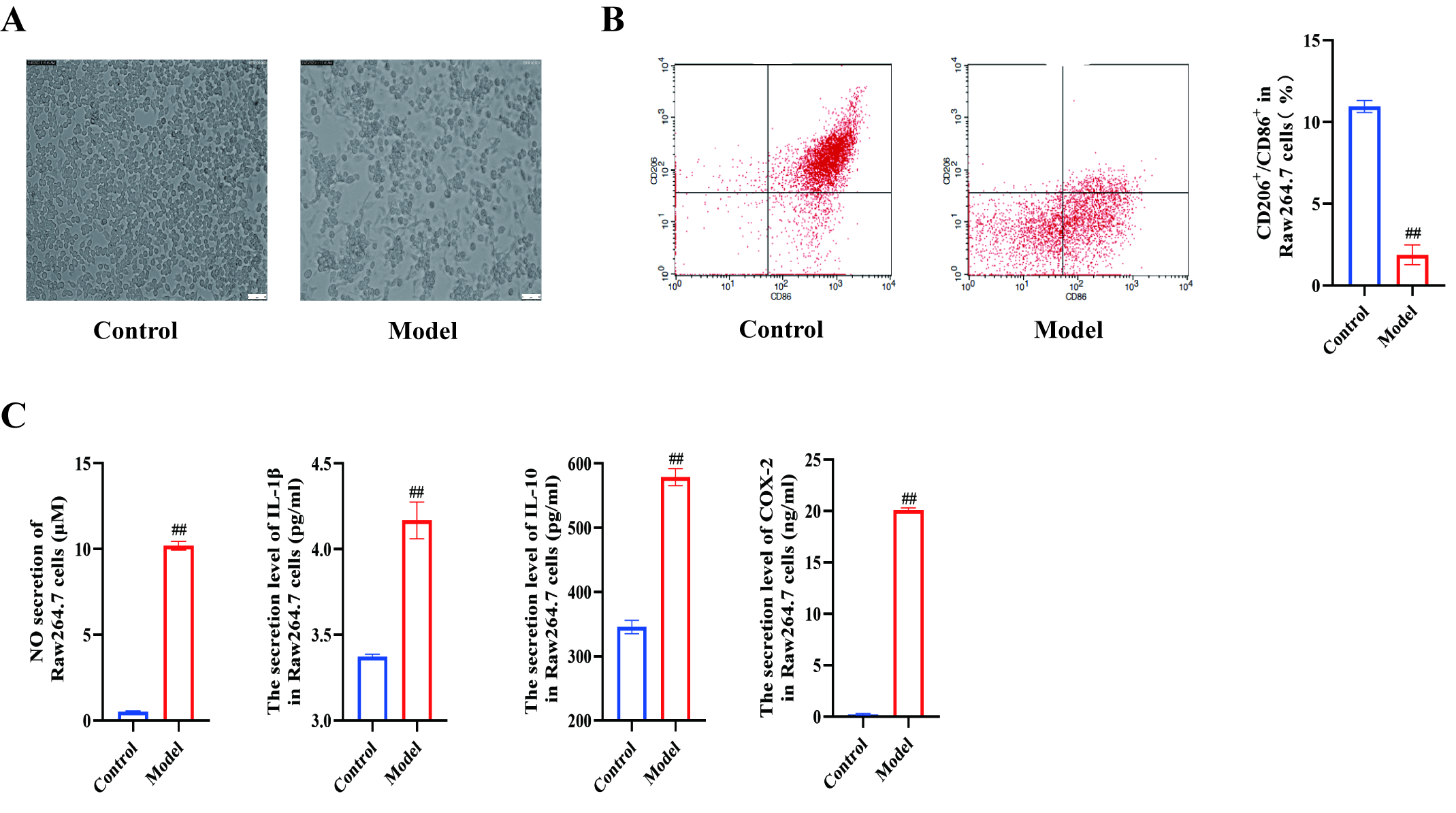
When using LPS to establish the Raw264.7 cell inflammation model, we used microscopic observation, flow cytometry and inflammatory factor detection to ensure that macrophages had undergone M1 polarization changes, as shown in the figure 1, we observed that after LPS stimulation, macrophages grew pseudopodia, the ratio of CD206+/CD86+ decreased significantly, and macrophages underwent M1 phenotypic transformation. Meanwhile, LPS significantly increased the secretion of inflammatory cytokines such as NO, IL-1β, IL-10, and COX-2 in Raw264.7 cells.

**Fig.S4** LPS promotes macrophage polarization. (A) Representative microscopic observations of Raw264.7 cells after no or LPS stimulation. (B) Flow cytometry evaluated M1 macrophages polarization by staining CD86/CD206. (C) NO concentration and the level of IL-1β, IL-10 and COX-2 in the supernatant of Raw264.7 cells after no or LPS stimulation.
